# A genome-wide association scan in admixed Latin Americans identifies loci influencing facial and scalp hair features

**DOI:** 10.1038/ncomms10815

**Published:** 2016-03-01

**Authors:** Kaustubh Adhikari, Tania Fontanil, Santiago Cal, Javier Mendoza-Revilla, Macarena Fuentes-Guajardo, Juan-Camilo Chacón-Duque, Farah Al-Saadi, Jeanette A. Johansson, Mirsha Quinto-Sanchez, Victor Acuña-Alonzo, Claudia Jaramillo, William Arias, Rodrigo Barquera Lozano, Gastón Macín Pérez, Jorge Gómez-Valdés, Hugo Villamil-Ramírez, Tábita Hunemeier, Virginia Ramallo, Caio C. Silva de Cerqueira, Malena Hurtado, Valeria Villegas, Vanessa Granja, Carla Gallo, Giovanni Poletti, Lavinia Schuler-Faccini, Francisco M. Salzano, Maria-Cátira Bortolini, Samuel Canizales-Quinteros, Francisco Rothhammer, Gabriel Bedoya, Rolando Gonzalez-José, Denis Headon, Carlos López-Otín, Desmond J. Tobin, David Balding, Andrés Ruiz-Linares

**Affiliations:** 1 Department of Genetics, Evolution and Environment, and UCL Genetics Institute, University College London, London WC1E 6BT, UK; 2 Departamento de Bioquímica y Biología Molecular, IUOPA, Universidad de Oviedo, Oviedo 33006, Spain; 3 Laboratorios de Investigación y Desarrollo, Facultad de Ciencias y Filosofía, Universidad Peruana Cayetano Heredia, Lima, 31, Perú; 4 Departamento de Tecnología Médica, Facultad de Ciencias de la Salud, Universidad de Tarapacá, Arica 1000009, Chile; 5 Roslin Institute and Royal (Dick) School of Veterinary Studies, University of Edinburgh, Midlothian EH25 9RG, UK; 6 Centro Nacional Patagónico, CONICET, Puerto Madryn U9129ACD, Argentina; 7 National Institute of Anthropology and History, México 4510, México; 8 GENMOL (Genética Molecular), Universidad de Antioquia, Medellín 5001000, Colombia; 9 Unidad de Genómica de Poblaciones Aplicada a la Salud, Facultad de Química, UNAM-Instituto Nacional de Medicina Genómica, México 4510, México; 10 Facultad de Medicina, UNAM, México 4510, México; 11 Departamento de Genética, Universidade Federal do Rio Grande do Sul, Porto Alegre 91501-970, Brasil; 12 Instituto de Alta Investigación, Universidad de Tarapacá, Arica 1000000, Chile; 13 Centre for Skin Sciences, Faculty of Life Sciences, University of Bradford, Bradford BD7 1DP, Victoria, UK; 14 Schools of BioSciences and Mathematics and Statistics, University of Melbourne, Melbourne 3010, Australia

## Abstract

We report a genome-wide association scan in over 6,000 Latin Americans for features of scalp hair (shape, colour, greying, balding) and facial hair (beard thickness, monobrow, eyebrow thickness). We found 18 signals of association reaching genome-wide significance (*P* values 5 × 10^−8^ to 3 × 10^−119^), including 10 novel associations. These include novel loci for scalp hair shape and balding, and the first reported loci for hair greying, monobrow, eyebrow and beard thickness. A newly identified locus influencing hair shape includes a Q30R substitution in the Protease Serine S1 family member 53 (*PRSS53*). We demonstrate that this enzyme is highly expressed in the hair follicle, especially the inner root sheath, and that the Q30R substitution affects enzyme processing and secretion. The genome regions associated with hair features are enriched for signals of selection, consistent with proposals regarding the evolution of human hair.

## Abstract (web summary)

By examining Latin American individuals of mixed European, Native American and African ancestry, Adhikari *et al*. identify novel loci influencing various features of facial and scalp hair. The study also provides experimental evidence that one of the implicated genes (PRSS53) is expressed in the hair follicle and that the top associated variant alters processing of this enzyme.

## Graphical abstract

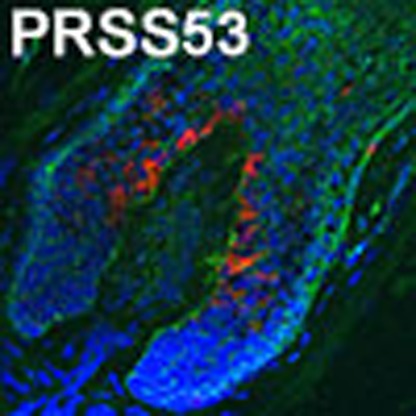

There is great diversity in primates with regard to hair appearance, including its distribution, shape and colour^1^. Hair plays a range of important functions, including thermal regulation, camouflage, sensory and social signalling, and the evolution of hair has been proposed to be influenced by both natural and sexual selection^1^. Humans differ from other primates in having lost most terminal body hair (via hair follicle miniaturization), possibly in connection to the adaptive development of more efficient sweating, linked to bipedalism^2^,^3^. However, considerable head hair has been retained in modern humans and its appearance shows extensive variation between individuals^4^,^5^,^6^. Head hair appearance is highly heritable^7^,^8^,^9^,^10^ and certain traits show high differentiation between continental native populations. For instance, variation in hair colour is essentially restricted to West Eurasia^11^, whereas straight hair is virtually absent from sub-Saharan Africa^4^. It has been proposed that variation in head hair appearance has been influenced by selection during the evolution of modern humans^2^,^3^,^11^. Although genetic association studies for human hair traits are few to date, loci influencing male pattern baldness, scalp hair colour and shape (curliness) have been identified in samples of European and East Asian ancestry^6^. Consistent with the key role of androgens in balding, the androgen receptor (*AR*) gene region has been highlighted as a major determinant among several loci associated with male-pattern baldness^12^. The genes associated with hair colour, involved in various aspects of melanocyte biology and melanin synthesis, also often impact on skin and eye pigmentation^13^. Interestingly, different genes have been associated with straight hair in Europeans and East Asians, suggesting that this trait evolved independently at least twice. The most robust associations for straight hair have implicated Trichohyalin (*TCHH*, a structural hair protein) in Europeans^14^,^15^, and *EDAR* (a cell signalling receptor) in East Asians^16^, illustrating the range of cellular mechanisms that can impact on hair shape.

To further our understanding of the genetic basis of variation in human hair, we performed a genome-wide association study (GWAS) in individuals of mixed European, Native American and African ancestry, who show high genetic diversity and extensive variation in head hair appearance. We report ten novel genetic associations, including the first reported loci for hair greying and for facial hair distribution/density. A newly identified locus influencing scalp hair shape encodes a serine protease (PRSS53) that we show is expressed in the hair follicle, with strongest association seen for a Q30R substitution that we show affects enzyme processing. Similar to what has been observed in the *EDAR* gene region, we find evidence of recent selection in East Asians at *PRSS53*.

## Results

### Study population and phenotypes

**Figure 1:**
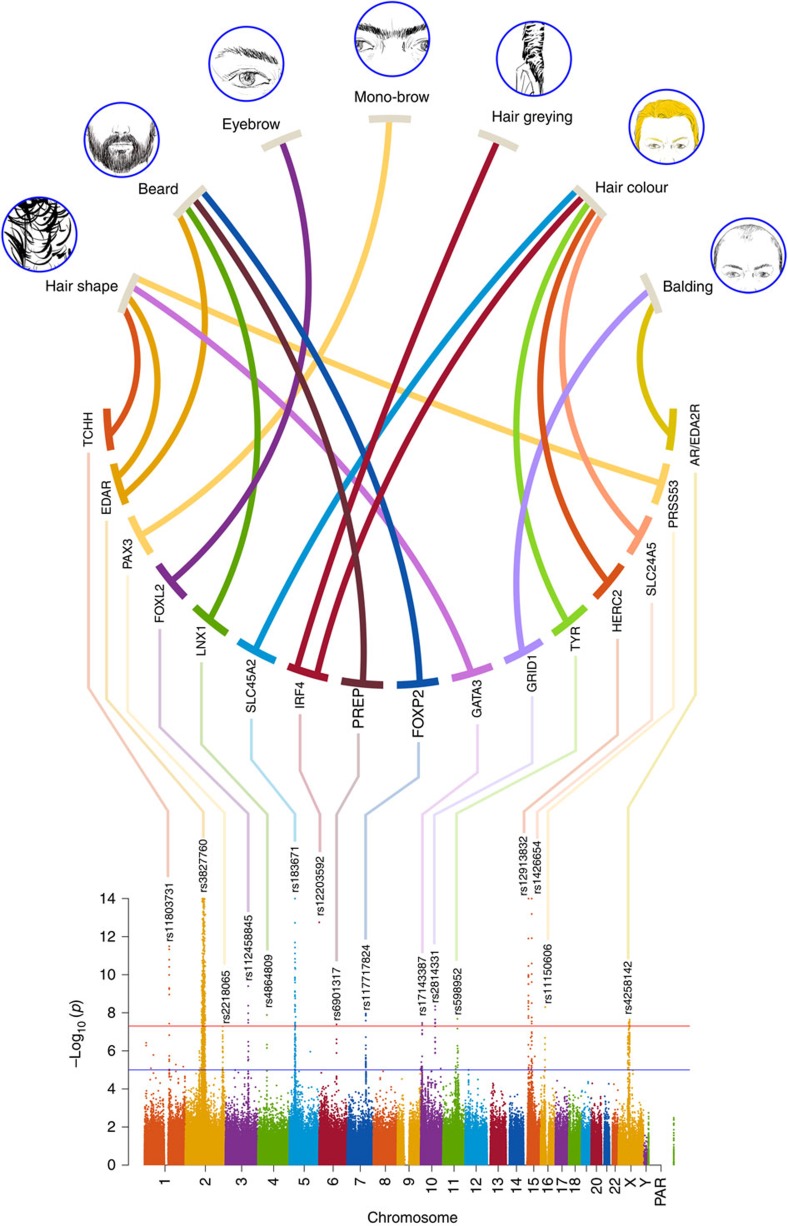
GWAS results overview. At the top are shown drawings illustrating the seven hair features examined in the CANDELA study sample. Thick lines connect these features with the candidate genes identified in regions with SNPs reaching genome-wide significant association (Table 1). At the bottom is shown a composite Manhattan plot displaying all significantly associated SNPs for the hair features examined. The rs number of the SNP with the smallest *P* value is shown at the top of each association peak (Table 1 index SNP). Composite panels in this and subsequent figures were made using Photoshop^67^.

Our study sample consists of 6,630 volunteers from the CANDELA cohort recruited in five Latin American countries (Brazil, Colombia, Chile, México and Perú; Supplementary Table 1)^17^,^18^. In these individuals, we performed a categorical assessment (in men and women) of: scalp hair shape (curliness), colour, balding and greying as well as (in men) of beard thickness (that is, density), monobrow and eyebrow thickness (Fig. 1 and Supplementary Fig. 1). These individuals were genotyped on Illumina's Omni Express BeadChip. After applying quality control filters, 669,462 single-nucleotide polymorphisms (SNPs) and 6,357 individuals were retained for further analyses (including 2,922 males). Average autosomal admixture proportions for this sample were estimated as: 48% European, 46% Native American and 6% African, but with substantial inter-individual variation (Supplementary Fig. 2). Several of the traits examined show low to moderate, but significant, correlations between them and with basic covariates (using a 10^−3^ Bonferroni-adjusted threshold; Supplementary Table 2). The highest significant trait correlations occur between: beard and eyebrow density and monobrow (*r*=0.14 to *r*=0.24); balding and beard density (*r*=0.15); hair greying and balding (*r*=0.13) and hair greying and beard density (*r*=0.13). The highest trait-covariate correlations detected were: age with hair greying (*r*=0.56), balding (*r*=0.15), beard density (*r*=0.28) and eyebrow thickness (*r*=−0.17); balding and hair colour with sex (*r*=−0.35 and *r*=−0.10); European ancestry correlates most strongly with hair colour (*r*=−0.32) and beard density (*r*=0.3). Based on a kinship matrix derived from the SNP data^19^, we estimated narrow-sense heritability using GCTA^20^. We found significant values for all the traits examined (Supplementary Table 3), with the highest heritability being estimated for hair colour (1) and the lowest for hair greying (0.27). These estimates of heritability are similar to other available estimates based on family data^7^,^8^,^9^,^10^.

### GWAS for hair features

**Figure 2:**
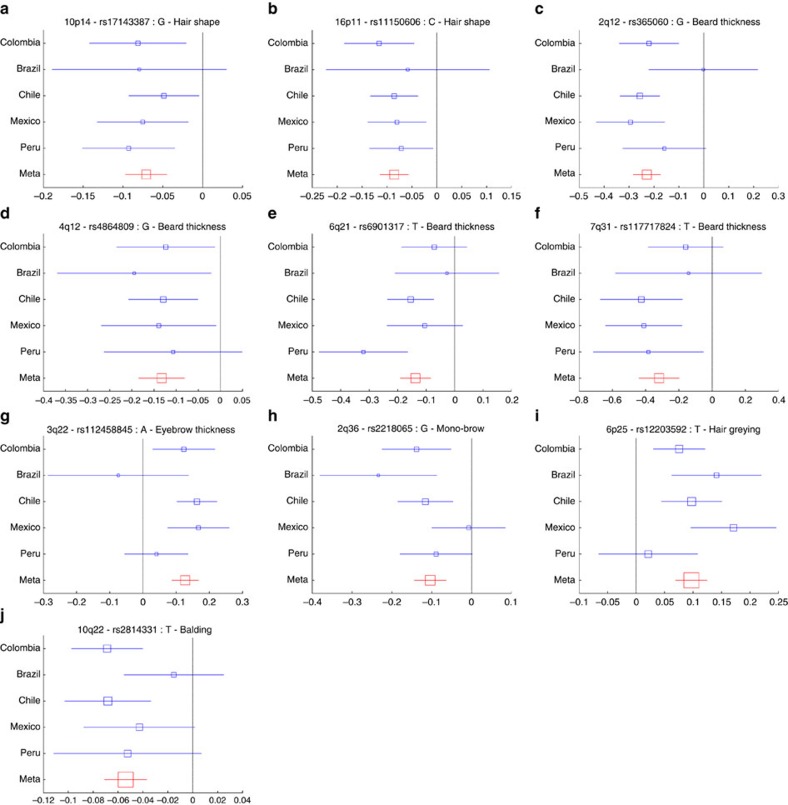
Effect sizes for the derived allele at index SNPs (Table 1) in ten genomic regions not previously associated with hair traits. (**a**) 10p14 hair shape, (**b**) 16p11 hair shape, (**c**) 2q12 beard thickness, (**d**) 4q12 bear thickness, (**e**) 6q12 beard thickness (**f**) 7q31 beard thickness (**g**) 3q22 eyebrow thickness, (**h**) 2q36 monobrow, (**i**) 6p25 hair greying, (**j**) 10q22 balding. Blue boxes represent regression coefficients (*x* axis) estimated in each country. Red boxes represent effect sizes estimated in the combined meta-analysis. Blue box sizes are proportional to sample size. Horizontal bars indicate a 95% confidence interval of width equal to 2 × standard errors. Meta-analysis *P* values are shown in Supplementary Table 4. Similar plots for regions previously associated with hair traits that were replicated here are shown in Supplementary Fig. 4.

We performed genome-wide association tests on 9,143,600 chip genotyped and imputed SNP data using multivariate linear regression, as implemented in PLINK^21^, using an additive genetic model adjusting for: age, sex and the first five SNP principal components (Supplementary Fig. 3). All the traits scored showed genome-wide significant association (*P* values<5 × 10^−8^) with SNPs in at least one genomic region (Fig. 1 and Table 1). We re-examined the association signals for each index SNP in every country sample separately (by performing independent association tests) and combined results as a meta-analysis using METAL^22^. For each SNP, significant effects were in the same direction in all countries, the variability of effect size across countries reflecting sample size (Fig. 2, Supplementary Fig. 4 and Supplementary Table 4). To probe the extent of phenotypic variation captured by the genetic data, we constructed a phenotype prediction model combining the index SNPs (Table 1) and a BLUP (Best Linear Unbiased Predictor) random effects component calculated from the genome-wide kinship matrix^19^, in addition to covariates (Supplementary Table 5). The BLUP term is sensitive to the effect of SNPs associated below the threshold for genome-wide significance. The prediction accuracy of this model was broadly consistent with the heritability estimates (Supplementary Table 3). Highest prediction accuracy was observed for hair colour, with 41% of the total phenotypic variance for this trait being captured by the model, including 20% explained by the index SNPs and the BLUP component. The lowest prediction accuracy was seen for monobrow, for which 16.2% of the total phenotypic variance was captured by the model. The difference between the heritability estimates and the prediction accuracy partly reflects limitations of the model, including that the BLUP component is likely to capture imperfectly the polygenicity of the traits examined (Supplementary Fig. 5).

### Candidate genes in genome regions associated with hair traits

**Figure 3:**
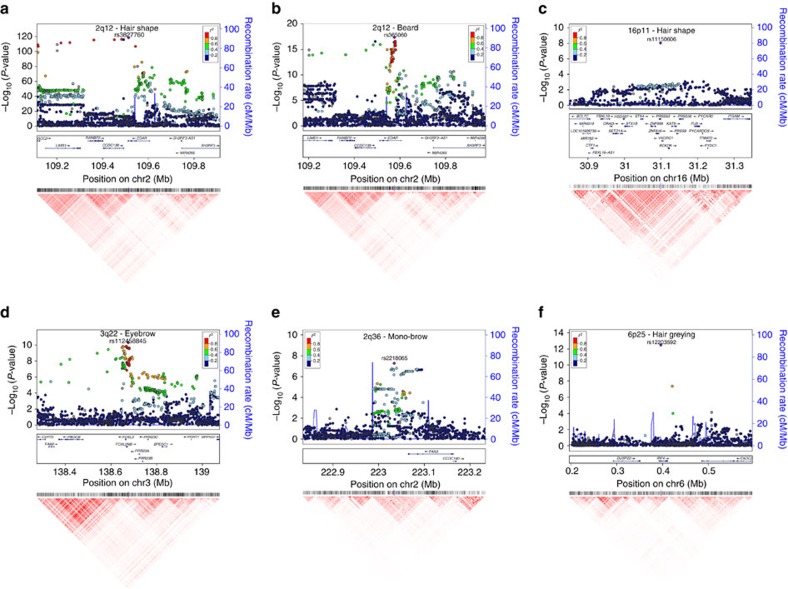
Association plots for six regions with SNPs showing genome-wide significant association to hair traits. (**a**) 2q12 hair shape, (**b**) 2q12 beard thickness, (**c**) 16p11 hair shape, (**d**) 3q22 eyebrow thickness, (**e**) 2q36 monobrow, (**f**) 6q25 hair greying. The index SNP in each region (Table 1) is shown as a purple diamond. At the top of the figure are shown the association results (on a -log_10_ *P* scale; left *y* axis) for all genotyped and imputed SNPs. The dot colour indicates the strength of LD (*r*^2^) between the index SNP and each SNP (based on the 1000genomes AMR data set). Recombination rate across the region, in the AMR data, is shown as a continuous blue line (scale on the right *y* axis). Genes in the region are shown in the middle. These plots were produced using LocusZoom^68^. Below each LocusZoom plot we show an LD heatmap (using *r*^2^, red indicating *r*^2^=1 and white indicating *r*^2^=0) produced using Haploview^69^. Coordinates used are from human genome sequence build 37. Plots for regions not shown here are presented in Supplementary Fig. 8.

**Figure 4:**
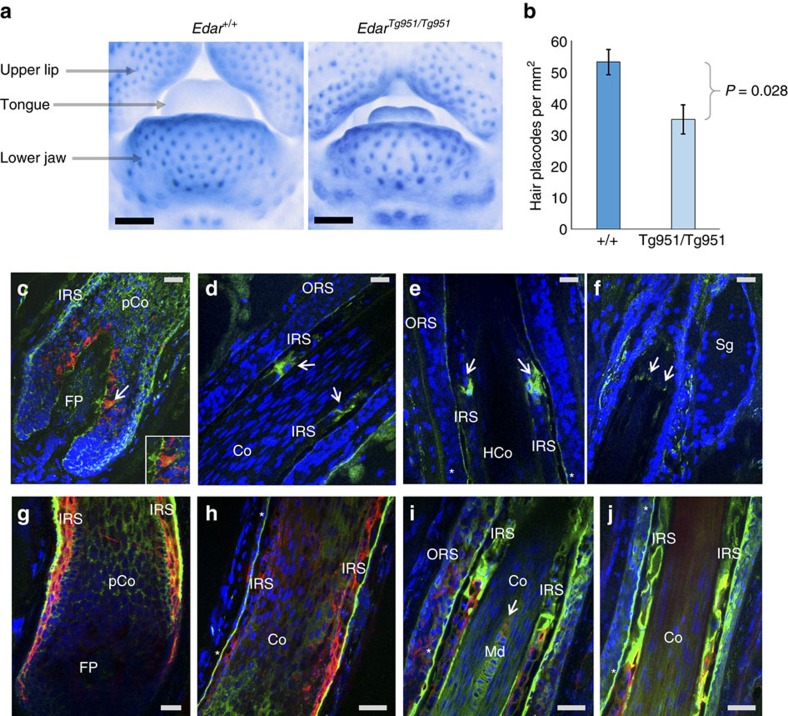
*EDAR* effects on mouse facial hair follicle density and expression of PRSS53 in anagen (growing) human hair follicles. (**a**) Frontal photographs showing part of the lower facial region from 14.5-day-old mouse embryos stained by *in-situ* hybridization for detecting *Sostdc1* to reveal hair placodes (primordia of hair follicles) as blue foci. We compared placode density in the lower jaw of wild-type (+/+) mice with an *Edar* transgenic having a high copy number of *Edar (Edar*^*Tg951/Tg951*^)^26^. The black scale bar equals 0.5 mm. (**b**) Bar plot comparing placode density in mice with different *Edar* genotypes (*n*=4). Mean density in *Edar*^*+/+*^ mice was 53 placodes per mm^2^ (standard deviation=1.3) and 35 placodes per mm^2^ (standard deviation=1.5) in *Edar*^*Tg951/Tg951*^ mice, the difference between means being significant (exact *P* value of 0.028). Error bars represent ±3 standard deviations. (**c**–**f**) Anagen human hair follicle stained with anti-PRSS53 antibody (green) and with anti-melanocyte antibody (red), and counterstained with 4,6-diamidino-2-phenylindole (DAPI; blue, nuclei). (**c**) Hair follicle bulb showing PRSS53 expression in the developing IRS, pre-cortex and in some melanocytes (arrow and inset) as indicated by the yellow–orange staining. (**d**) Mid hair follicle showing expression of PRSS53 in maturing IRS keratinocytes (arrows). (**e**) Distal hair follicle showing high expression of PRSS53 in IRS cells around the level of DNA degradation in hair fibre (HF) keratinocytes (as indicated by a reduction in DAPI staining in this region HF). PRSS53 is also expressed in the IRS companion layer (CL; *). (**f**) Upper distal hair follicle at sebaceous gland level (Sg) showing PRSS53 expression in scattered peri-follicular cells just below the Sg and in the IRS at the point of its dissolution (arrows). (**g**–**j**) Anagen human hair follicle stained with anti-PRSS53 antibody (green) and with anti-TCHH antibody (red), and counter-stained with DAPI (blue, nuclei). (**g**) Hair follicle bulb showing co-localization (orange/yellow) of PRSS53 and TCHH in the developing IRS, especially in the most external IRS layer. (**h**) Supra-bulbar region of the hair follicle showing expression of PRSS53 in the developing companion layer (*) of the IRS (green) and some co-localization with TCHH in the inner IRS. (**i**) Mid hair follicle showing expression of PRSS53 in the IRS companion layer (*) and the central medulla (Md) of the developing HF. (**j**) Upper hair follicle showing PRSS53 expression in the companion layer of the IRS (*) and in the TCHH-positive IRS. Co, hair fibre cortex; FP, follicular papilla; IRS, inner root sheath; Md, medulla; pCo, pre-cortex; ORS, outer root sheath; Sg, sebaceous gland. Grey scale bars correspond to 40 μm in each figure.

Several of the regions showing genome-wide significant association include strong candidate genes. Scalp hair shape and beard thickness are strongly associated with SNPs in 2q12 (Fig 3a,b) and two other traits (eyebrow thickness and monobrow) show genome-wide suggestive *P* values for SNPs in this same region (Supplementary Fig. 6). Associated SNPs overlap the *EDAR* (ectodysplasin A receptor) gene. EDAR acts as part of the EDA-EDAR-EDARADD signalling pathway^23^ during prenatal development to specify the location, size and shape of ectodermal appendages, such as hair follicles, teeth and glands^23^. Strongest association with hair shape was observed for SNP rs3827760 (Fig. 3a and Table 1), coding for a V370A substitution in EDAR. This variant has been robustly associated with hair shape in East Asians^16^,^24^,^25^. In a previous study of the CANDELA sample, which examined 30 ancestry informative markers, we found association between hair shape and a SNP in *EDAR* that is in strong linkage disequilibrium (LD) with rs3827760 in the 1000 genomes data^17^. The *EDAR* SNP showing strongest association with beard density is not the coding SNP rs3827760 associated with hair shape but rather rs365060 located ∼62 kb upstream of rs3827760 in the first intron of *EDAR* (Fig. 3b). Several SNPs in this intron have smaller association *P* values than rs3827760 and analyses conditioning on rs3827760 suggest that the association signal of SNPs in this intron is independent from that observed at rs3827760 (Supplementary Fig. 7). These intronic SNPs are located in a different LD block and there is a recombination hotspot between them and rs3827760 (Fig 3a,b and Supplementary Fig. 7A). Interestingly, the first intron of *EDAR* is rich in regulatory elements and SNPs in this intron show evidence of recent selection in Europeans (discussed further below). Hypohidrotic ectodermal dysplasia is a Mendelian disorder caused by mutations in the EDA-EDAR-EDARADD pathway and is characterized by sparse scalp hair, eyebrows and eye lashes^23^. Transgenic mice with increased *Edar* function have been shown to have thickened and straightened hair fibres^26^,^27^. We therefore examined chin hair follicle density in wild-type mice and in an *Edar* gain-of-function transgenic strain (*Edar*^*Tg951/Tg951*^)^26^. Consistent with the effect of *EDAR* on beard thickness, we found that the *Edar*^*Tg951/Tg951*^ strain has significantly lower chin hair follicle density compared with wild-type mice (Fig. 4a,b).

Apart from 2q12, scalp hair shape is associated with SNPs in three other genomic regions (in 1q21, 10p14 and 16p11). Genome-wide significant association in the 16p11 region was strongest for rs11150606, the derived allele of which codes for a Q30R substitution in the Protease Serine S1 family member 53 (PRSS53; Fig. 3c and Table 1). Proteases and protease inhibitors are known to be important for epidermal keratinization and regulate hair growth and cycling^28^. A spontaneous mouse mutant (frizzy, *fr*), characterized by curly whiskers, carries an amino-acid substitution in another serine protease (Prss8)^29^ and a conditional knock-out with no expression of *Prss8* in the epidermis shows hair abnormalities in newborns and defects in corneocyte morphogenesis, epidermal lipid composition, profilaggrin processing and tight junction assembly^30^. The 1q21.3 region overlaps the trichohyalin gene (*TCHH*), which has been previously associated with scalp hair curliness in Europeans^14^,^15^. Strongest association was seen for SNP rs11803731, encoding a M790L substitution in TCHH (Supplementary Fig. 8), consistent with previous GWASs^14^,^15^. *TCHH* is expressed in cornifying keratinocytes of epithelia, particularly in the inner root sheath (IRS) and the hair fibre medulla of hair follicles where it is involved in the cross-linking of the cornified envelope with cellular keratin filaments^31^. SNPs in 10p14 overlap LINC00708, ∼150 kb downstream of the GATA-binding protein 3 gene (*GATA3*), an interesting candidate in this region (Supplementary Fig. 8). *Gata3* is expressed in the hair follicle IRS of mice and a *Gata3* null mutant shows abnormal hair growth and shape^32^. Interestingly, *Gata3* mutant hair follicles have greatly reduced expression of trichohyalin^33^.

Eyebrow thickness shows genome-wide significant association to SNPs on 3q23 overlapping the forkhead box L2 (*FOXL2*) gene (Fig. 3d). Rare mutations in the *FOXL2* gene region (including coding variants as well as upstream and downstream intergenic rearrangements) cause blepharophimosis syndrome (BPES)^34^, an autosomal dominant eyelid malformation often accompanied by thick eyebrows. Mouse experiments have shown that *Foxl2* is expressed around the eyes up to the time that hair is formed (E13.5)^35^ and a mutant with altered *Foxl2* expression (and BPES features) typically shows hair loss around the eyes^36^.

Monobrow shows genome-wide significant association with SNPs in 2q36 with strongest association being observed for marker rs2395845 located ∼70 kb downstream of the paired box gene 3 (*PAX3*) gene, an interesting candidate in the region (Fig. 3e). Rare mutations of *PAX3* have been shown to cause Waardenburg syndrome type 1 (WS1). WS is a clinically and genetically heterogeneous Mendelian disorder of neural crest derivatives whose manifestations include deafness, a range of pigmentation abnormalities, broad nasal bridge and monobrow (seen in ∼85% of WS1 patients^37^). Intronic SNPs within *PAX3* have been implicated in recent GWASs of facial morphology, particularly in relation with nasion position (the point just above the nasal bridge)^38^,^39^. PAX3 is a key transcription factor during embryogenesis and analysis of mouse mutants have confirmed that it is essential to guide normal development of neural crest derivatives^40^.

Hair greying shows genome-wide significant association to SNP rs12203592 in intron 4 of the interferon regulatory factor 4 gene (*IRF4*; Fig. 3f). This SNP also shows association with hair colour in our sample (Table 1) and in previous studies this SNP has been associated with skin, hair and eye pigmentation^13^. Recent *in-vitro* studies have shown that rs12203592 impacts on the function of an enhancer element regulating *IRF4* expression and the induction of tyrosinase (TYR), a key enzyme in melanin synthesis^41^. In addition to *IRF4*, hair colour (but not hair greying) shows genome-wide significant association to four other well-established pigmentation gene regions (*SLC45A2*, *TYR*, *OCA2*/*HERC2* and *SLC24A5*; Table 1 and Supplementary Fig. 8)^13^. Finally, for balding, we replicate association to the well-established Androgen Receptor/Ectodysplasin A2 Receptor (*AR/EDA2R*) locus on Xq12 (ref. 12; Supplementary Fig. 8).

The other four genomic regions showing genome-wide association include no strong candidate genes with established roles in hair biology (Table 1 and Supplementary Fig. 8). Beard thickness is associated with SNPs in 7q31, 4q12 and 6q21 where the nearest genes are, respectively: forkhead box P2 (*FOXP2*), ligand of numb-protein X 1 (*LNX1*) and prolyl endopeptidase (*PREP*; Supplementary Fig. 8). None of these genes have known functions specifically related to hair. Similarly, balding is associated with SNPs in the second intron of the Glutamate receptor delta-1 subunit gene (*GRID1*) on 10q22 but this gene has no documented role in hair biology.

### PRSS53 and hair shape

Among the SNPs in regions showing genome-wide significant association, a functional role potentially underlying the observed association is most suggestive for rs11150606 encoding the R30Q substitution in PRSS53, associated with scalp hair shape. Bioinformatic analysis indicates that this amino-acid change could introduce a subtilisin/kexin-like proprotein convertase site in PRSS53 (Supplementary Fig. 9), and thus could have the potential of affecting processing of the enzyme. To probe into the role of PRSS53 in hair development, we performed immunohistochemistry of human scalp hair follicles during active hair growth (anagen phase; Fig. 4c–j). Expression of PRSS53 was mainly detected in the developing IRS and pre-cortex of the hair fibre and in some bulbar melanocytes (Fig. 4c). PRSS53 expression was increased in subpopulations of IRS keratinocytes corresponding to early and late stages of hair fibre keratinization and IRS cornification (Fig. 4d,e). This pattern of expression is consistent with the hair-shape association we observe, as the cornifying IRS is thought to impart significant hair-shaping influence on the hair fibre^42^. Expression of PRSS53 appears to be modulated as a function of hair fibre differentiation, as evidenced by high PRSS53 expression at the level of the hair follicle where the hair fibre undergoes dissolution of nuclear DNA (Fig. 4e)^43^. Double immunofluorescence for PRSS53 and TCHH revealed co-expression of these proteins in specific elements of the IRS (Fig. 4g–j), suggesting that PRSS53 may also be associated with the maturation of this hair follicle sheath. The only other component of the hair follicle that expresses TCHH is the medulla of the hair fibre and this was also found to express PRSS53 (Fig. 4i). The medulla is a non-obligate component of the hair fibre, but when present it impacts on the mechanical properties of hair^44^. It is interesting that PRSS53 is expressed in the companion layer of the IRS (Fig. 4e,h–j), directly opposing the non-keratinizing, non-upwardly moving outer root sheath. It is not yet known how the ‘slippage' of the inner versus outer hair follicle components occurs during outward hair fibre growth, but it is likely that protease activity is involved. Expression of PRSS53 changes in the upper IRS (Fig. 4f), at the level of the sebaceous gland, where the IRS undergoes a controlled dissolution, required for exiting of the hair fibre to the skin surface.

**Figure 5:**
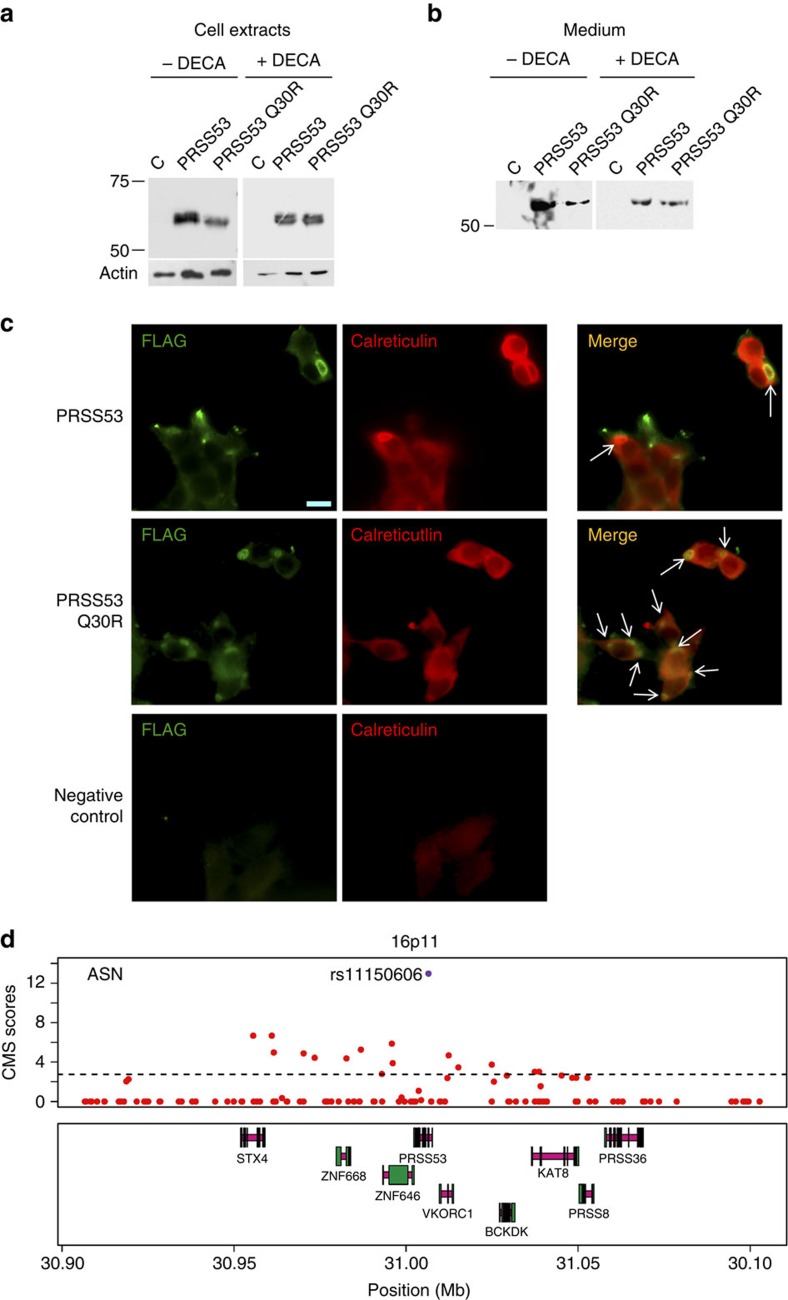
Processing of PRSS53 and signals of selection in the PRSS53 gene region. Comparison of PRSS53 and PRSS53 (Q30R) from cell extracts (**a**) and media (**b**), after expression in 293-EBNA cells cultured in the absence (−) or presence (+) of DECA (decanoyl-RVKR-CMK, a pro-protein convertase inhibitor). (**a**) The top two bands seen in lanes loaded with PRSS53 cell extracts, result from partial processing of the signal peptide. In the absence of DECA, there appears to be an accumulation of PRSS53 (Q30R) without signal peptide. PRSS53 (Q30R) also has a slightly faster mobility compared with PRSS53 (a difference of eight amino acids based on the location of the pro-protein convertase site Supplementary Fig. 9). In the presence of DECA, both forms of the enzyme appear identically processed and there is no difference in mobility of the proteins. (**b**) The medium from cell cultures grown in the absence of DECA shows a reduced amount of PRSS53 (Q30R), compared with PRSS53. In the presence of DECA, the amount of protein in the media is similar for the two forms of the enzyme. Recombinant proteins were detected using an anti-FLAG antibody after migration on 13% SDS–polyacrylamide gel electrophoresis gels. Molecular weight markers (kDa) are indicated on the left. Beta-actin was used as a loading control (C=cells transfected with an empty vector). Full immunoblots for **a** and **b** are shown in Supplementary Fig. 11. (**c**) Immunostaining of 293-EBNA cells expressing PRSS53 and PRSS53 (Q30R). The enzyme was detected using an anti-FLAG antibody and the endoplasmic reticulum (ER) stained with an anti-calreticulin antibody. There is more abundant co-localization of the enzyme with the ER (white arrows) for PRSS53 (Q30R) compared with PRSS53, consistent with the intracellular accumulation of PRSS53 (Q30R). Negative controls, lacking primary antibodies for FLAG and calreticulin, are also shown. The scale bar indicates 10 μm. Magnification was the same for all photographs. (**d**) The top panel shows CMS scores (red dots) in the 1000 genomes ASN (JPT+CHB) data for SNPs in the 16p11 region associated with hair shape. The dotted line indicates the empirical significance threshold (1%). The SNP with the highest CMS score is rs11150606, coding for the Q30R substitution in PRSS53 (highlighted in purple) associated with hair curliness (Table 1 and Fig. 3c). Genes in the region are shown in the panel below (introns in magenta, exons in green). Coordinates are from human genome sequence Build 37.

To evaluate the cellular impact of the Q30R substitution in PRSS53, we expressed both forms of the enzyme in 293-EBNA cells, a human cell line showing pro-protein convertase activity^45^. Western blot analysis of transfected cell extracts revealed a different signal peptide processing in the two forms of the enzyme and that PRSS53 Q30R has a slightly faster electrophoretic mobility, consistent with an extra proteolytic cleavage (Fig. 5a and Supplementary Fig. 9). These differences were not detected when the cell cultures were incubated with an inhibitor of pro-protein convertases (decanoyl-RVKR-CMK, DECA). Consistent with an altered processing of the enzyme affecting its secretion, the PRSS53 Q30R variant was less abundant in the cell culture media, except when cells were incubated in the presence of DECA (Fig. 5b). In agreement with the reduced secretion of PRSS53 Q30R seen in the western blot analyses, immunocytochemistry indicates a greater accumulation of this variant in the ER-transGolgi network (Fig. 5c). Altogether, these *in-vitro* analyses confirm that the Q30R substitution in PRSS53 can affect processing and secretion of the enzyme.

### Signatures of selection at associated gene regions

Some of the strongest signals of selection in the human genome detected in recent genome-wide searches involve pigmentation genes in Europeans and *EDAR* in East Asians^27^,^46^. To assess whether selection could have contributed broadly to shape variation at gene regions impacting on human hair features, we examined whether there is enrichment for signatures of selection at the regions showing evidence of association with hair traits in the CANDELA sample. For this purpose, we used the Composite of Multiple Signals (CMS) statistic calculated in the three main reference East Asian, European and African populations from the 1000 Genomes Project data^47^ (ASN, CEU and YRI, respectively). We contrasted the distribution of CMS scores at gene regions showing at least suggestive association to hair features (that is, regions marked by SNPs with association *P* values<10^−5^; Supplementary Table 6) with the distribution of CMS scores across the genome. We found significantly higher CMS scores in the hair-associated gene regions compared with the genome-wide distribution (one-sided Mann–Whitney *U*-test *P* value=2 × 10^−8^, *P*=2 × 10^−5^ and *P*=2 × 10^−5^ in ASN, CEU and YRI, respectively) and a significantly higher proportion of SNPs with empirically significant CMS scores (that is, SNPs in the top 1% of the distribution; one-sided Mann–Whitney *U*-test *P* values of 5 × 10^−7^, 1 × 10^−4^ and 4 × 10^−4^, in ASN, CEU and YRI, respectively). Noticeably, significant CMS scores were observed in ASN for SNPs in the *PRSS53* region associated with hair shape, the highest CMS score (12.96, empirical *P* value 3 × 10^−4^) being observed for SNP rs11150606, encoding the Q30R substitution in PRSS53 (Fig. 5d). In the *EDAR* region, we observe the previously documented strong signal of selection in ASN for variants around SNP rs3827760 (refs 27, 46, associated with hair shape (Table 1). We also observe a significant signal of selection in CEU for the intronic *EDAR* variants associated here with beard thickness, independently of rs3827760, discussed above (Fig. 3b and Supplementary Fig. 7b).

## Discussion

The analyses presented here have enabled us to expand substantially the set of gene regions known to impact on variation in human head hair appearance. This task has been facilitated by the extensive phenotypic and genetic diversity of the CANDELA sample, a result of Latin American history involving admixture between Africans, Europeans and Native Americans^17^. The predominant European and Native American ancestry of the CANDELA sample is expected to provide especially high power for the detection of genetic effects at loci with differentiated allele frequencies between those two continental populations. Consistently, allele frequencies at most index SNPs identified here show large differences between Europeans and East Asians/Native Americans (Supplementary Table 7). This strong differentiation in allele frequencies could relate partly to selection acting on the associated gene regions, as proposed for the evolution of human hair appearance^2^,^3^,^11^. The enrichment we observe for significant CMS scores at gene regions associated with hair features is consistent with this scenario. Interestingly, the pattern of variation at *PRSS53* is similar to that seen for *EDAR*, with significant CMS scores in East Asians and a functional amino acid changing variant reaching high frequency only in East Asia (Supplementary Table 7). These observations, and the fact that other genes are associated with straight hair in Europeans, are in line with the proposal that scalp hair shape has been the subject of recent selection in humans^2^,^3^.

There is increasing interest in elucidating the mechanisms influencing the shape of the growing hair fibre. Based on mouse studies it has been proposed that EDAR signalling is involved in determining hair shape through regulating expression of the key hair growth signal Shh (Sonic hedgehog)^26^. Higher Edar function increases *Shh* expression and causes it to become symmetrically expressed in the hair bulb, leading to straight hair growth presumably through promotion of symmetric cell proliferation^26^. Structurally, a key player in shaping hair is TCHH, involved in crosslinking the cornified envelope and keratins of IRS cells^31^. TCHH is one of the earliest differentiation proteins of the growing (anagen) hair follicle bulb, and is enriched in the IRS (representing ∼30% of its protein content). TCHH confers significant mechanical strength to the IRS via its enrichment in arginines and glutamines, which represent over 40% of TCHH amino acids. Many of the arginine residues undergo citrullination/deimination, whereas the glutamines are involved in intra- and interprotein chain crosslinks during the cornification/hardening of the IRS^31^. The progressive hardening of the IRS is thus thought to contribute to the moulding of the still-pliable hair fibre during hair growth. It is interesting to note that the TCHH SNP with smallest *P* value observed here (rs11803731) has also been strongly implicated by previous analyses in Europeans^15^,^48^, suggesting that this variant might be directly affecting hair shape. The M790L substitution in TCHH encoded by rs11803731 is not predicted to result in major structural alterations of TCHH, but it has been proposed that it could affect the post-translational processing of TCHH, with regulatory implications^48^. It will be important to assess whether TCHH is a substrate for PRSS53, given our observation that this enzyme is enriched in all layers of the IRS during the cornification/hardening stages of the hair fibre, as well as in the medulla (a component influencing the physico-mechanical characteristics of the hair fibre^6^). Proteases and protease inhibitors are important for epidermal keratinization and can regulate hair growth and cycling^28^. For example, optimal desquamation of the IRS (and overall hair growth cycling) is perturbed in lysosomal cysteine protease cathepsin L knock-out mice^49^. Moreover, given the similarity of PRSS53 with the kallikreins (the largest family of secreted serine protease endopeptidases) it is possible that PRSS53 lyses substrates in cornified tissues that ultimately desquamate^50^. Interestingly, it has been suggested that GATA3 (in the 10p14 region associated with hair shape) inhibits the expression of serine protease inhibitor Kazal type-5 (SPINK5), mutations of which cause Netherton syndrome, an autosomal recessive congenital ichthyosis characterized by so-called Bamboo hair, epidermal hyperplasia and an impaired epidermal barrier function^51^.

The colour of hair results from melanin pigments transferred to hair fibre keratinocytes from hair follicle melanocytes. These melanocytes differentiate from melanoblasts that migrate from the neural crest into hair follicles early in development^6^. Some hair follicle melanoblasts remain undifferentiated and serve as stem cells for the periodic replenishment of mature melanocytes, melanogenesis occurring only in the anagen phase of the hair growth cycle. Among the several hundred gene products known to participate in melanogenesis, recent association studies have identified a handful that influence hair colour variation in Europeans^13^. Most of these associations have been replicated here (Table 1), including that of the derived T allele at SNP rs12203592 in *IRF4* with lighter hair colour. It has been shown experimentally that IRF4 interacts with the microphthalmia-associated transcription factor (MITF, a key regulator of the expression of many pigment enzymes and differentiation factors), to activate the expression of TYR (a rate-limiting essential enzyme in melanin synthesis). The derived T allele at rs12203592 leads to reduced TYR expression and melanin synthesis, consistent with the association of this allele with lighter hair colour^41^. In line with the geographic distribution of light hair colour, the T allele at rs12203592 is essentially absent outside Europe (Supplementary Table 7). Interestingly, we find that the T allele at SNP rs12203592 is also associated with increased hair greying. Experimental evidence suggests that the mechanism of hair greying involves incomplete maintenance of melanocyte stem cells in the hair follicle^6^. Importantly, MITF is known to affect melanocyte survival via its regulation of anti-apoptotic Bcl2 expression, a key factor in protection of the hair follicle against oxidative stress^6^. To probe the mechanism by which IRF4 might impact on hair greying, it will therefore be important to evaluate whether the T allele at rs12203592 influences MITF in terms of melanoblast stem cell maintenance and survival or via melanocyte loss post differentiation.

The skin on different parts of the body has different hair characteristics, with the final hair distribution dependent upon the spacing pattern laid down during development, the extent of skin growth that occurs subsequent to pattern establishment, and to hormonal and ageing effects. The development of skin at different body sites is known to be controlled by an underlying transcription factor code^52^ to which FOXL2, FOXP2 and PAX3 may contribute in defining hair distribution on specific areas of the face. As hair of the beard is produced through a two-stage process of embryonic hair follicle patterning followed by a post-pubertal androgen-driven transformation into terminal hair, beard thickness could be modulated by genes acting either prenatally or at puberty. Our finding of reduced hair placode density in embryonic mice with increased *Edar* expression suggests that the basis for this variation lies in the recognized role of EDAR in developmental hair patterning^53^. It is likely that EDAR function affects hair follicle density on most or all of the human body, as described in the mouse^26^, but that on the head this effect is most readily apparent as variation in beard thickness. Analyses focusing on the mechanism by which associated genetic variants affect regional facial hair density should provide insights into developmental patterning in humans, and perhaps yield clues into the genetic basis for the striking modification of hair distribution that has occurred in human evolution^54^.

Elucidating the genetic architecture of normal variation in hair traits has implications outside basic bioscience. Among human visible phenotypes hair appearance is perhaps the mostly easily modified, a feature prominently exploited by the cosmetics industry. This industry has traditionally focused on the development of products altering the appearance of keratinous hair fibres after their exit from the skin surface. However, there is currently great interest in exploring whether hair appearance can be modified as it is formed in the hair follicle^55^. This includes evaluating whether hair greying could be slowed or blocked, and elucidating the mechanism by which IRF4 influences hair greying could provide targets of intervention for this purpose. Similarly, modulating the activity of PRSS53 in the IRS and medulla is a candidate pathway with a view to purposefully altering hair shape. The genetics of hair appearance is also of interest in anthropology and forensics, particularly for the prediction of hair features based on genetic information. The implementation of this so-called ‘forensic DNA phenotyping' promises to contribute an investigative tool in cases where a biological sample is available but there is lack of other information regarding the identity of its contributor. The development of this approach in Europeans is fairly advanced for hair colour, and is beginning to be explored for balding and hair shape^56^. Considering the high genetic and phenotypic diversity of Latin American populations, appropriate tools will need to be in place for reliable phenotypic prediction in that context and the results presented here represent a step in that direction.

## Methods

### Study subjects

A total of 6,630 volunteers from five Latin American countries (Brazil, Chile, Colombia, Mexico and Peru), part of the CANDELA consortium sample (http://www.ucl.ac.uk/silva/candela)^17^,^18^, were included in this study (Supplementary Table 1). Ethics approval was obtained from: the Universidad Nacional Autónoma de México (México), the Universidad de Antioquia (Colombia), the Universidad Peruana Cayetano Heredia (Perú), the Universidad de Tarapacá (Chile), the Universidade Federal do Rio Grande do Sul (Brazil) and the University College London (UK). All participants provided written informed consent. Blood samples were collected by a certified phlebotomist and DNA extracted following standard laboratory procedures.

### Hair phenotyping

Scalp hair features were recorded by physical examination of the volunteers. Natural hair colour was scored in four categories (1-red/reddish, 2-blond, 3-dark blond/light brown or 4-brown/black). Greying was scored on a five-point scale: 1-for no greying, 2-for predominant non-greying, 3-for ∼50% greying, 4-for predominant greying and 5-for totally white hair. Hair curliness was scored as 1-straight, 2-wavy, 3-curly or 4-frizzy. Balding was scored on a three-point scale (none, medium, high) in both women and men. Although frequency of balding in women is low, their inclusion more than doubles sample size, thus adding considerable power to the analyses (Supplementary Fig. 10). Facial hair traits were scored using photographs of the faces of the individuals. Beard density was scored in men using a three-point scale (low, medium or high), separately for shaven and unshaven individuals and scores for these two groups subsequently merged. As an interview of the volunteers indicated that most women modified their eyebrows, monobrow and eyebrow thickness were also only scored in men. Both these traits were scored on three-point scales: eyebrow thickness as low, medium or high, and monobrow as none, medium or high.

The frequency distribution of the traits in the CANDELA sample analysed here is shown in Supplementary Fig. 1.

### DNA genotyping and quality control

DNA samples from participants were genotyped on the Illumina HumanOmniExpress chip including 730,525 SNPs. PLINK v1.9 (ref. 57) was used to exclude SNPs and individuals with more than 5% missing data, markers with minor allele frequency <1%, related individuals and those who failed the X-chromosome sex concordance check (sex estimated from X-chromosome heterozygosity not matching recorded sex information). After applying these filters, 669,462 SNPs and 6,357 individuals (2,922 males and 3,435 females) were retained for further analysis. Because of the admixed nature of the study sample (Supplementary Fig. 2), there is an inflation in Hardy–Weinberg *P* values. We therefore did not exclude markers based on Hardy–Weinberg deviation.

### Statistical genetic analyses

*P* values for Pearson correlation coefficients were obtained by permutation. Narrow-sense heritability (computed as the additive phenotypic variance explained by a kinship matrix computed from the chip genotypes) was estimated using GCTA^20^ by fitting an additive linear model with a random effect term whose variance is given by the kinship matrix, with age and sex as covariates. The kinship matrix was obtained using the LDAK approach^19^, which accounts for LD between SNPs. African, European and Native American ancestry was estimated from a set of 93,328 autosomal SNPs (LD-pruned from the full chip data) via supervised runs of ADMIXTURE^58^. Reference putative parental population data included in the ADMIXTURE analyses for Africans and Europeans were chosen from HAPMAP and for Native Americans from selected Amerindian populations as described in Ruiz-Linares *et al*.^17^

The chip genotype data were phased using SHAPEIT2 (ref. 59). IMPUTE2 (ref. 60) was then used to impute genotypes at untyped SNPs using variant positions from the 1000 Genomes Phase I data^61^. The 1000 Genomes reference data set included haplotype information for 1,092 individuals for 36,820,992 variant positions. Positions that are monomorphic in 1000 Genomes Latin American samples (CLM, MXL and PUR) were excluded, leading to 11,025,002 SNPs being imputed in our data set. Of these, 22,737 SNPs had imputation quality scores <0.3 and were excluded. The IMPUTE2 genotype probabilities at each locus were converted into best-guess genotypes using PLINK^57^. SNPs with uncalled genotypes in >5% of samples or minor allele frequency <1% were excluded. The final imputed data set used in the GWAS analyses included genotypes for 9,143,600 SNPs.

PLINK 1.9 (ref. 57) was used to perform genome-wide association tests for each phenotype using multiple linear regression with an additive genetic model incorporating age, sex and five genetic PCs (principal components) as covariates. The genetic PCs were obtained from the LD-pruned data set of 93,328 SNPs using PLINK 1.9. These PCs were selected by inspecting the proportion of variance explained and checking scatter and screen plots (Supplementary Fig. 3A). Individual outliers were removed and PCs recalculated after each removal. Using these PCs, the *Q*–*Q* plots for all association tests showed no sign of inflation, the genomic control factor *λ* being<1.02 in all cases (Supplementary Fig. 3B). We previously showed that using five PCs in GWAS of the CANDELA sample completely removes the inflation, which is observed when PC adjustment is not used, and increasing the number of PCs included in the regression from five to ten does not provide additional gain^18^. Association analysis on the imputed data set were performed using the best-guess imputed genotypes in PLINK and using the IMPUTE2 genotype probabilities obtained in SNPTEST v2.5 (ref. 62). Association results from both approaches were consistent with each other and with the results from the chip genotype data. For analysis of the X chromosome, an inactivation model was used (male genotypes encoded as 0/2 and female genotypes as 0/1/2). Individuals with red hair were excluded from the final hair colour GWAS, as it was a rare phenotype (0.55%). Similarly, few individuals were scored as having frizzy hair (2.4%) and these individuals were excluded from the hair shape GWAS. Analyses for hair greying were performed with the five-point scores or with a two-point scale of some greying or no greying and produced similar results.

A meta-analysis was carried out for the index SNPs identified in the GWAS (Table 1) by testing for association separately in each country sample and combining the results using the meta-analysis software METAL^22^ (as implemented in PLINK 1.9). Forest plots were produced with MATLAB combining all regression coefficients and standard errors. Cochran's *Q* statistic was computed for each trait to test for effect size heterogeneity across country samples. For SNPs with significant heterogeneity, a random effects model was used for meta-analysis^63^.

A prediction model was constructed for each hair trait examined, including the associated index SNPs (Table 1) as fixed effects and a random effect term obtained via BLUP (Best Linear Unbiased Predictor) using the genome-wide kinship matrix obtained from the chip data^19^. The BLUP component is thus sensitive to the effect of SNPs below the threshold for genome-wide significance. Prediction results were obtained through tenfold cross-validation^64^: the set of samples were randomly split into ten chunks of 10% and used as the test data set, whereas the remaining 90% was used as training data set to fit a prediction model. For each run, *R*^2^ estimates were calculated (conditional on the covariates) to obtain the proportion of phenotypic variance explained by the model. Average prediction scores across the tenfold runs were calculated. Genetic PCs, age and sex (except for facial hair traits which are male-only) were used as covariates.

To evaluate an enrichment of selection signals at gene regions associated with hair traits, we examined the CMS scores of selection calculated for the three main 1000 Genomes Project populations^46^: ASN (JPT+CHB), CEU and YRI. We obtained empirical significance cutoffs (1%) separately for each population based on CMS scores for 3,071,032, 3,179,944 and 3,312,050 SNPs (in ASN, CEU and YRI, respectively). In each population, we estimated the mean CMS score for SNPs in a ±2 kb region around each gene, based on the UCSC RefSeq annotation (excluding gene regions with less than four SNPs). We obtained the distribution of mean CMS scores for genes including SNPs associated with the hair traits examined here and compared it with the distribution for all other genes in the genome (19,835, 20,093 and 20,386 gene regions in ASN, CEU and YRI, respectively). We used a suggestive significance level cutoff for inclusion in the set of hair-associated genes (that is, SNP association *P* value <10^−5^; Table 1 and Supplementary Table 6), resulting in 53, 53 and 55 gene regions being included for ASN, CEU and YRI, respectively. If associated SNPs were in intergenic regions, we included the gene most closely located to this SNP. We contrasted the distribution of mean CMS scores at these gene regions with the distribution for all other gene regions in the genome using a one-sided Mann–Whitney *U*-test.

### Assessment of mouse chin hair follicle density

We examined chin hair placode (follicle primordium) density in E14.5 day mouse embryos. We focused on embryonic day 14.5 (E14.5) as at this embryonic stage the hair pattern is laid out, so that the sites of future hair follicle growth are readily quantifiable by detecting focal expression of the marker gene *Sostdc1* (ref. 65). We performed *in-situ* hybridization^18^ staining embryos for *Sostdc1* to visualize the placodes present on the lower jaw. At this stage, placodes are visible as rings or filled rings of *Sostdc1* expression. Embryos were positioned to view the lower jaw, imaged from a frontal view and placodes were counted within a rectangular area on the lower jaw. Placode density for each embryo was determined using Image-Pro software (Media Cybernetics).

Average hair placode density for the *Edar*^*+/+*^ was 53 placodes per mm^2^ (standard deviation=1.3, sample size=4) compared with *Edar*^*Tg951/Tg951*^, which was reduced to 35 placodes per mm^2^ (standard deviation=1.5, sample size=4). All values in the wild-type group were higher than in the transgenic group. A non-parametric Mann–Whitney *U*-test was applied to test the difference between the two sets of hair density values. An exact *P* value calculation was used.

### Immunohistochemistry of PRSS53

Unshaven, full-thickness human adult scalp with terminal hair was used snap frozen in liquid nitrogen in cubes of 2 cm^3^. Cryosections of 6–8 μm were cut using a cryostat onto adhesive glass slides and incubated with anti-PRSS53 antibody (NBP1, #90678 from Novus) and anti-trichohyalin antibody (AE15, # IQ337 from Immuquest) using standard double immunofluorescence protocols. IgG isotype controls were used at the same concentration as the smallest primary antibody dilution. Co-distribution and co-localization of both antigens in the hair follicle were determined by merging of the PRSS53- and trichohyalin-positive channels.

### *In-vitro* analysis of PRSS53

*Mutagenesis*. To introduce the Q30R substitution in the PRSS56 sequence, the QuikChange II XL Site-Directed Mutagenesis Kit (Agilent) was employed following the instructions by the manufacturer. Primers designed for mutagenesis were: poli3MUT-FOR 5′- CAGCGTGCCTGTGGACGGCGTGGCCCCGGC -3′ and poli3MUR-REV 5′- GCCGGGGCCACGCCGTCCACAGGCACGCTG -3′, and we employ as template a previously published plasmid containing the entire PRSS53 sequence, which includes a FLAG epitope^66^. PCR conditions were as follows: 95 °C, 1 min (1 cycle); 95 °C, 50 s, 66°, 50 s and 68° 12 min (18 cycles); and 68 °C, 7 min (1 cycle). PCR products were visualized in a 1.0% agarose gel and DNA sequence was verified before experimental use.

*Cell culture and transfection*. 293-EBNA cells were routinely maintained at 37 °C in 5% CO_2_ in DMEM supplemented with 10% fetal bovine serum and 50 μg ml^−1^ streptomycin and 100 U ml^−1^ penicillin (Life Technologies). Expression vectors were transfected into cells using TransIT-X2 Dynamic Delivery System (Mirus) as recommended by the manufacturer. Cell conditioned medium was obtained from 293-EBNA cultures for 2 days in medium without added serum. When indicated, the proprotein convertase inhibitor decanoyl-RVKR-CMK was added at 20 μmol L^−1^.

*Polyacrylamide gel electrophoresis and western blot*. Cell extracts were resolved by 13% polyacrylamide gel electrophoresis, transferred to a nitrocellulose membrane and then incubated overnight with an anti-FLAG antibody (Sigma). Immunoreactive proteins were visualized using horseradish peroxidase (HRP)-peroxidase-labelled anti-rabbit antibody (Pierce), and developed with the Luminata Forte Western HRP substrate (Millipore). An anti-actin antibody (Sigma) was employed to ascertain equal loading. To detect the presence of recombinant proteins in conditioned medium, 4 ml of conditioned medium were concentrated till 100 μl using and Speed-Vac centrifuge (Savant). Then, 12 μl of each sample were loaded per lane for further identification as indicated above. A pCEP control plasmid (empty vector) was used as a negative control for all these assays.

*Cell staining*. For immunocytochemical analysis of 293-EBNA cells expressing PRSS53 or PRSS53 Q30R, cells were fixed with 4% paraformaldehyde in phosphate-buffered saline buffer and cells were then blocked with 15% fetal bovine serum. To detect recombinant PRSS53 and PRSS53 Q30R proteins, blocked slides were incubated overnight with an anti-FLAG antibody (Sigma), followed by 2 h of incubation with a secondary Alexa 488-conjugated antibody (Life Technologies). An anti-calreticulin antibody (Mirus) was employed to examine co-localization of both forms of PRSS53 with endoplasmic reticulum. To detect calreticulin, a secondary Alexa 546-conjugated antibody was employed. For negative controls, same protocol was employed with the exception that primary antibodies were absent. Images were obtained using a fluorescence microscope and a digital camera (Axiovert).

## Additional information

**How to cite this article:** Adhikari, K. *et al*. A genome-wide association scan in admixed Latin Americans identifies loci influencing facial and scalp hair features. *Nat. Commun.* 7:10815 doi: 10.1038/ncomms10815 (2016).

## Supplementary Material

Supplementary Information Supplementary Figures 1-11, Supplementary Tables 1-7 and Supplementary References

## Figures and Tables

**Table 1:** Features of index SNPs showing strongest genome-wide significant association (*P* value <5 × 10^−8^) to the scalp and facial hair features examined in the CANDELA sample.

**Trait**	**Region**	**SNP**	**Closest gene**	**P value**	**Alleles ancestral>derived**
Hair shape	1q21	rs11803731	***TCHH***	3 × 10^−12^	A>T
Hair shape	2q12	rs3827760	***EDAR***	3 × 10^−119^	A>G
Hair shape	10p14	rs17143387	*GATA3*	4 × 10^−8^	T>G
Hair shape	16p11	rs11150606	***PRSS53***	7 × 10^−9^	T>C
Beard thickness	2q12	rs365060	***EDAR***	1 × 10^−15^	G>C
Beard thickness	4q12	rs4864809	*LNX1*	1 × 10^−8^	A>G
Beard thickness	6q21	rs6901317	*PREP*	4 × 10^−8^	G>T
Beard thickness	7q31	rs117717824	*FOXP2*	1 × 10^−8^	G>T
Eyebrow thickness	3q22	rs112458845	*FOXL2*	4 × 10^−10^	G>A
Monobrow	2q36	rs2218065	*PAX3*	5 × 10^−8^	A>G
Hair greying	6p25	rs12203592	***IRF4***	3 × 10^−13^	C>T
Hair colour	5p13	rs183671	*SLC45A2*	2 × 10^−59^	T>G
Hair colour	6p25	rs12203592	*IRF4*	1 × 10^−13^	C>T
Hair colour	11q14	rs598952	*TYR*	2 × 10^−8^	T>A
Hair colour	15q13	rs12913832	***HERC2/OCA2***	5 × 10^−104^	A>G
Hair colour	15q21	rs1426654	*SLC24A5*	1 × 10^−18^	G>A
Balding	10q22	rs2814331	***GRID1***	4 × 10^−9^	C>T
Balding	Xq12	rs4258142	*AR/EDA2R*	2 × 10^−8^	C>T

SNP, single-nucleotide polymorphism.

Regions with intragenic index SNPs are shown in bold (the gene name is underlined if this index SNP is coding). SNPs in regions reaching only genome-wide suggestive association, *P* value <10^−5^, are listed in Supplementary Table 6.
