# Supplementary material for: A genome-wide association scan in admixed Latin Americans identifies loci influencing facial and scalp hair features
Source: Nat Commun. 2016 Mar 1;7:10815. doi: 10.1038/ncomms10815 (PMC4773514; doi:10.1038/ncomms10815)
Supplement: Supplementary Information — Supplementary Figures 1-11, Supplementary Tables 1-7 and Supplementary References [file ncomms10815-s1.pdf]

SUPPLEMENTARY FIGURES

Supplementary Figure 1: Frequency distribution of hair traits in the CANDELA sample (N=6,630)

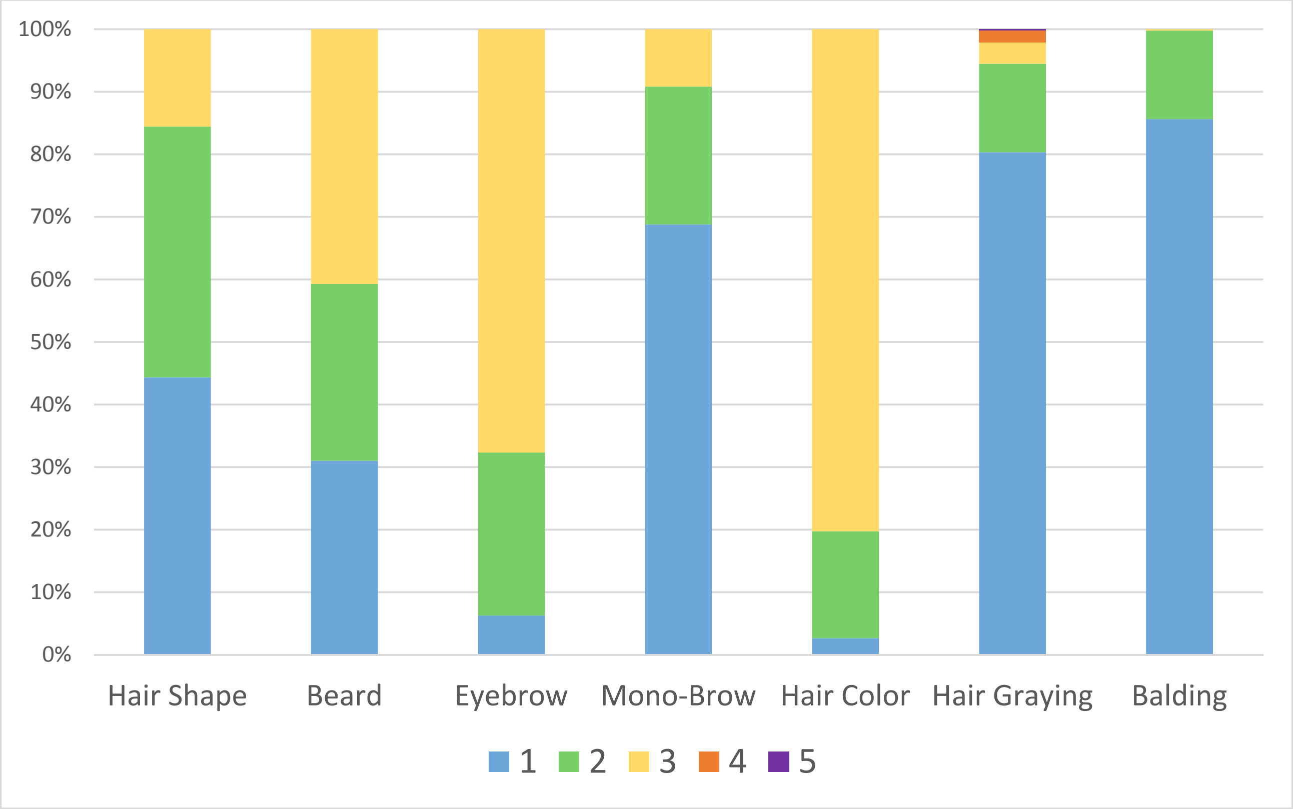

Hair trait	Category 1	Category 2	Category 3	Category 4	Category 5
**Hair shape**	Straight	Wavy	Curly	-	-
**Beard**	Low	Medium	High	-	-
**Eyebrow**	Low	Medium	High	-	-
**Monobrow**	None	Medium	High	-	-
**Hair color**	Blond	Dark blond/Light brown	Brown/Black	-	-
**Hair graying**	No graying	Predominantly no graying	50% graying	Predominant graying	Totally white hair
**Balding**	None	Medium	High	-	-

Supplementary Figure 2: Estimated African, European and Native American ancestry (%) in the CANDELA individuals included in the GWAS for hair traits (N=6,357)

Individual ancestry barplots for each country are shown below. Individuals within each country are sorted by increasing European ancestry.

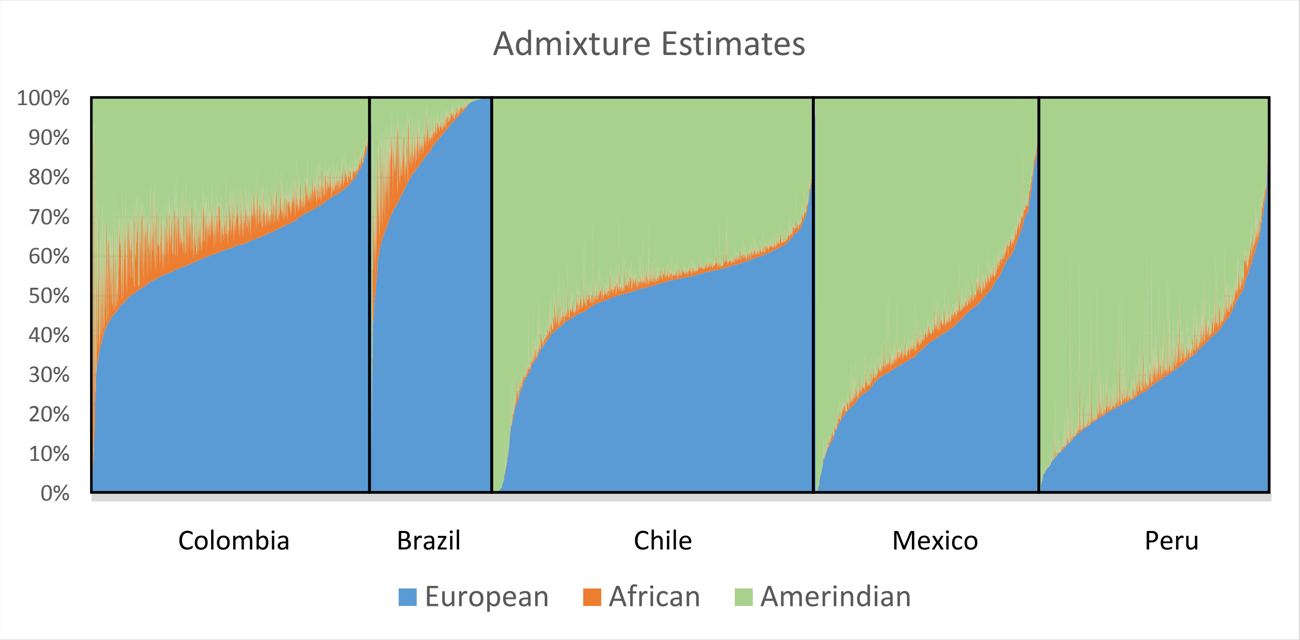

Mean ancestry estimates for each country and overall:

Country	African	European	Native American
Colombia	10%	61%	29%
Brazil	9%	79%	12%
Chile	5%	46%	49%
Mexico	5%	38%	58%
Peru	5%	31%	65%
Overall	6%	48%	46%

Supplementary Figure 3: Selection of genetic Principal Components for inclusion in the GWAS analyses

A) Scree plot:

Principal components (x-axis below) were extracted from an LD-pruned SNP dataset (see methods). The proportion of genetic variance explained by each PC is shown below (y-axis).

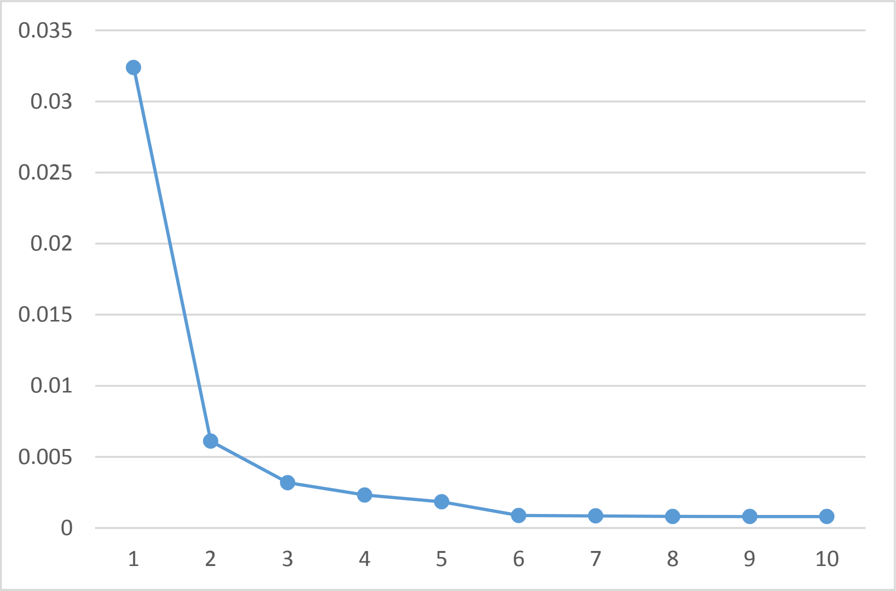

B) GWAS Q-Q plot:

Example Q-Q plot from the GWAS for hair graying obtained after inclusion of the top 5 genetic PCs as covariates. No residual inflation due to population substructure is observed (SNPs with high *P* values lie exactly on the diagonal relating observed and expected values). Genomic inflation factor λ = 1.007.

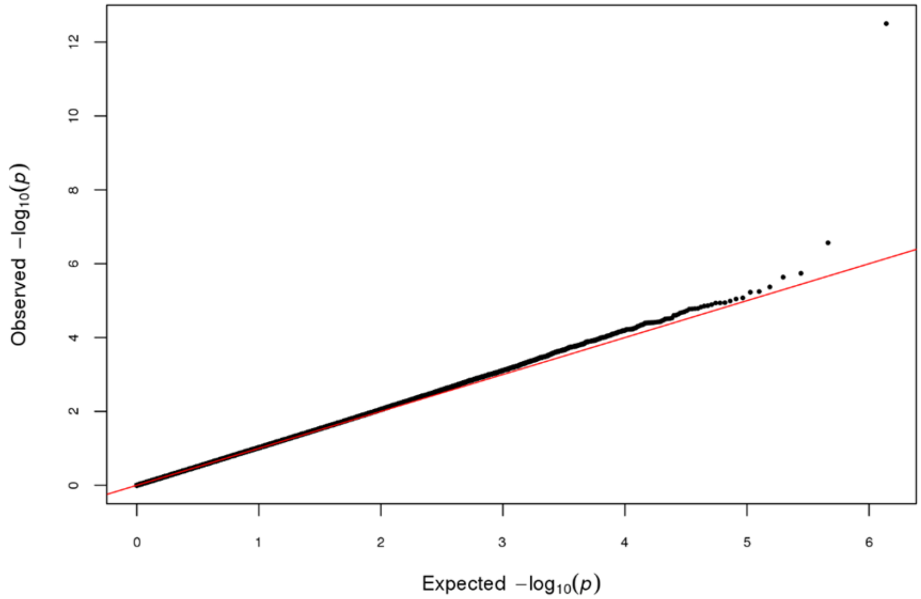

Supplementary Figure 4: Effect sizes for index SNPs in genomic regions previously associated with hair traits which were replicated here and are not shown in Figure 2.

Blue boxes represent regression coefficients (x-axis) estimated in each country. Red boxes represent effect sizes estimated in the combined meta-analysis. Box sizes are proportional to sample size. Horizontal bars indicate standard errors. Meta-analysis *P* values are shown in Supplementary Table 4.

a) 1q21 – Hair Shape

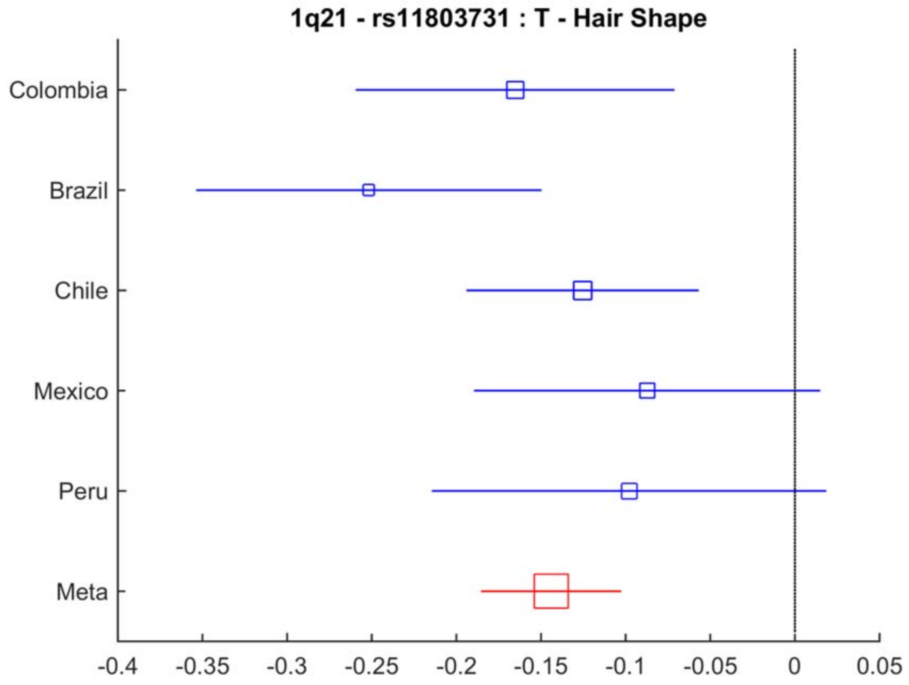

b) 2q12 – Hair Shape

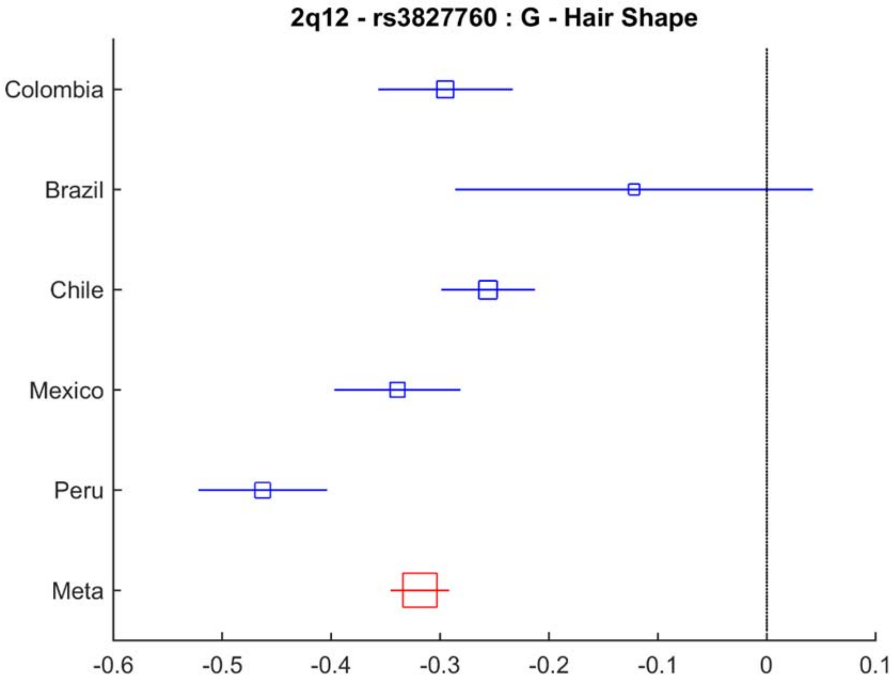

c) 5p13 – Hair Color

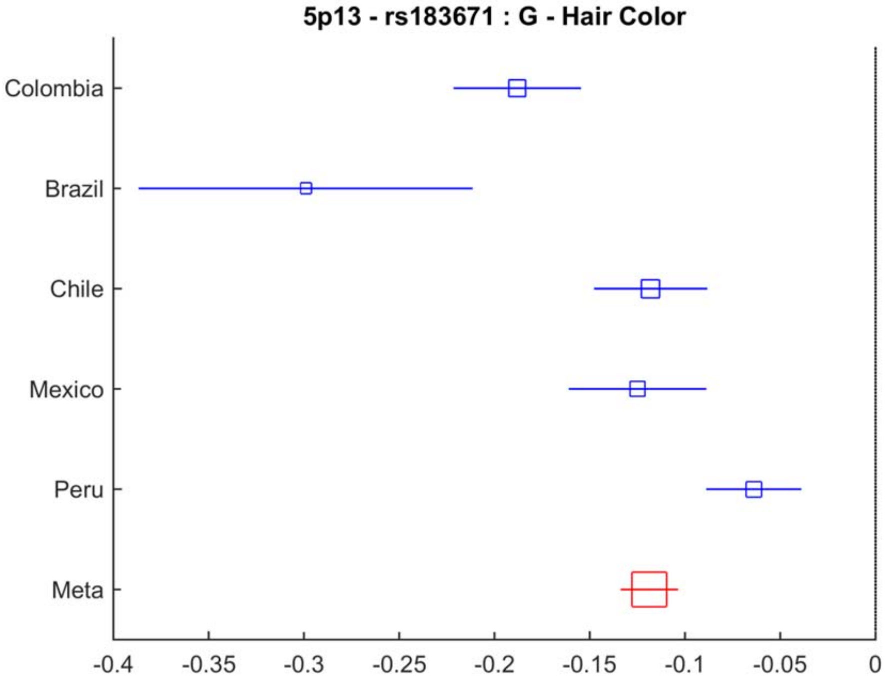

d) 6p25 – Hair Color

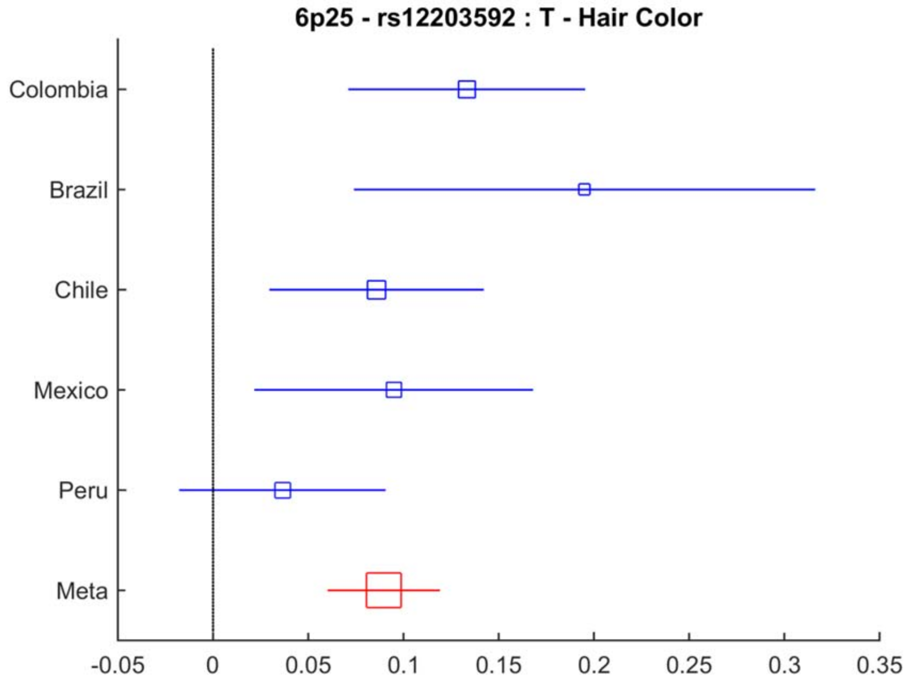

e) 11q14 – Hair Color

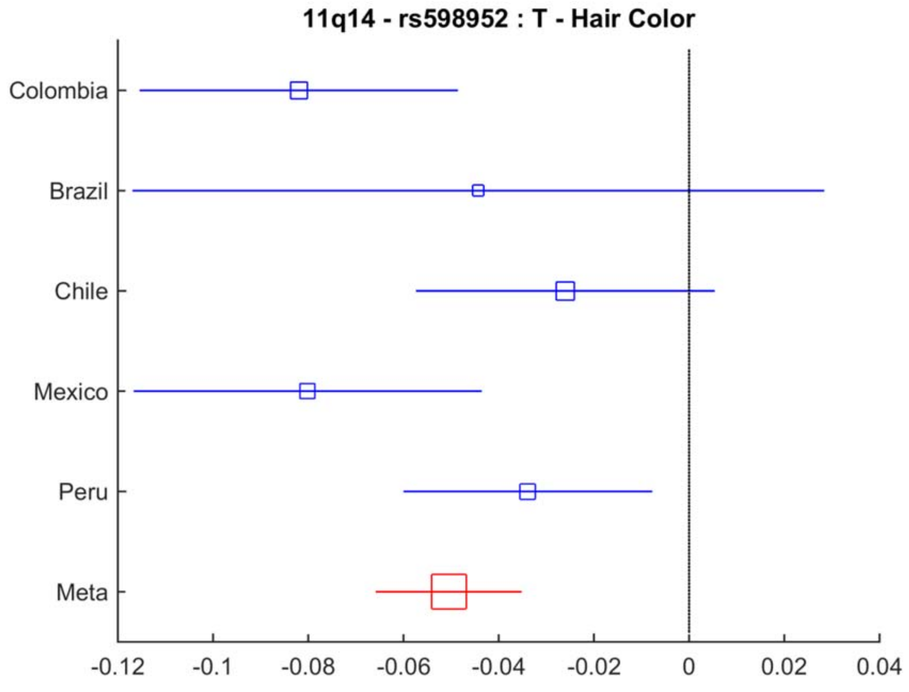

f) 15q13 – Hair Color

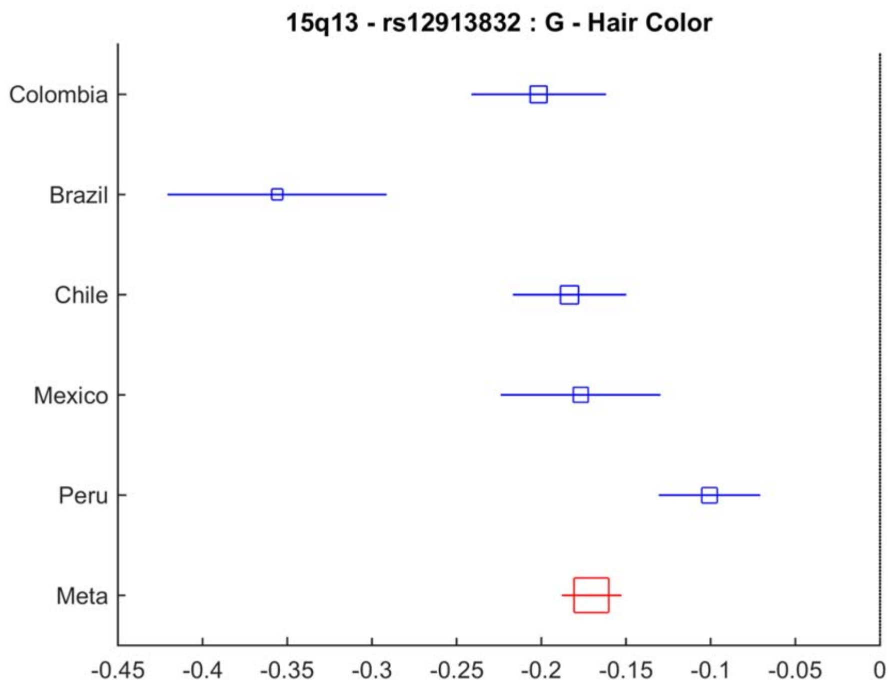

g) 15q21 – Hair Color

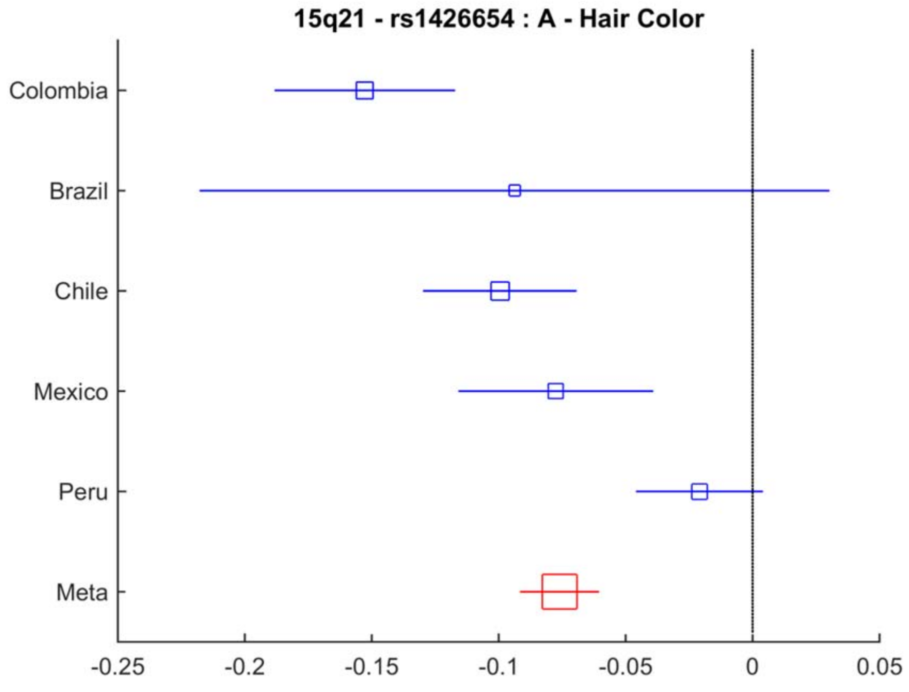

h) Xq12 – Balding

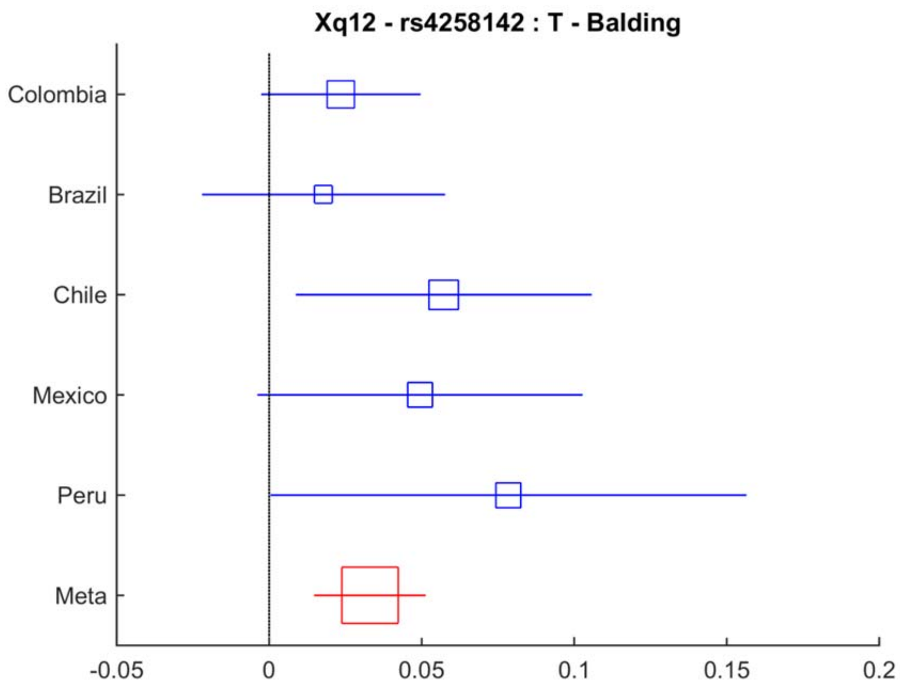

Supplementary Figure 5: Polygenic architecture of hair traits

For all hair traits there is no residual inflation of association *P* values, after correction for population substructure, a conclusion supported by all traits having genomic control inflation factor $\lambda < 1.02$. Also, in Q-Q plots SNPs with high *P* values lie exactly on the diagonal relating observed and expected values (Supplementary Figure 3B). However, hair traits can have a large number of SNPs with low *P* values deviating from the diagonal at the tail, suggesting that there are many SNPs with smaller effect sizes which are associated with the trait, albeit not reaching the genome-wide significance threshold of 5×10^{-8}. As an example, the Q-Q plot for hair color is shown below.

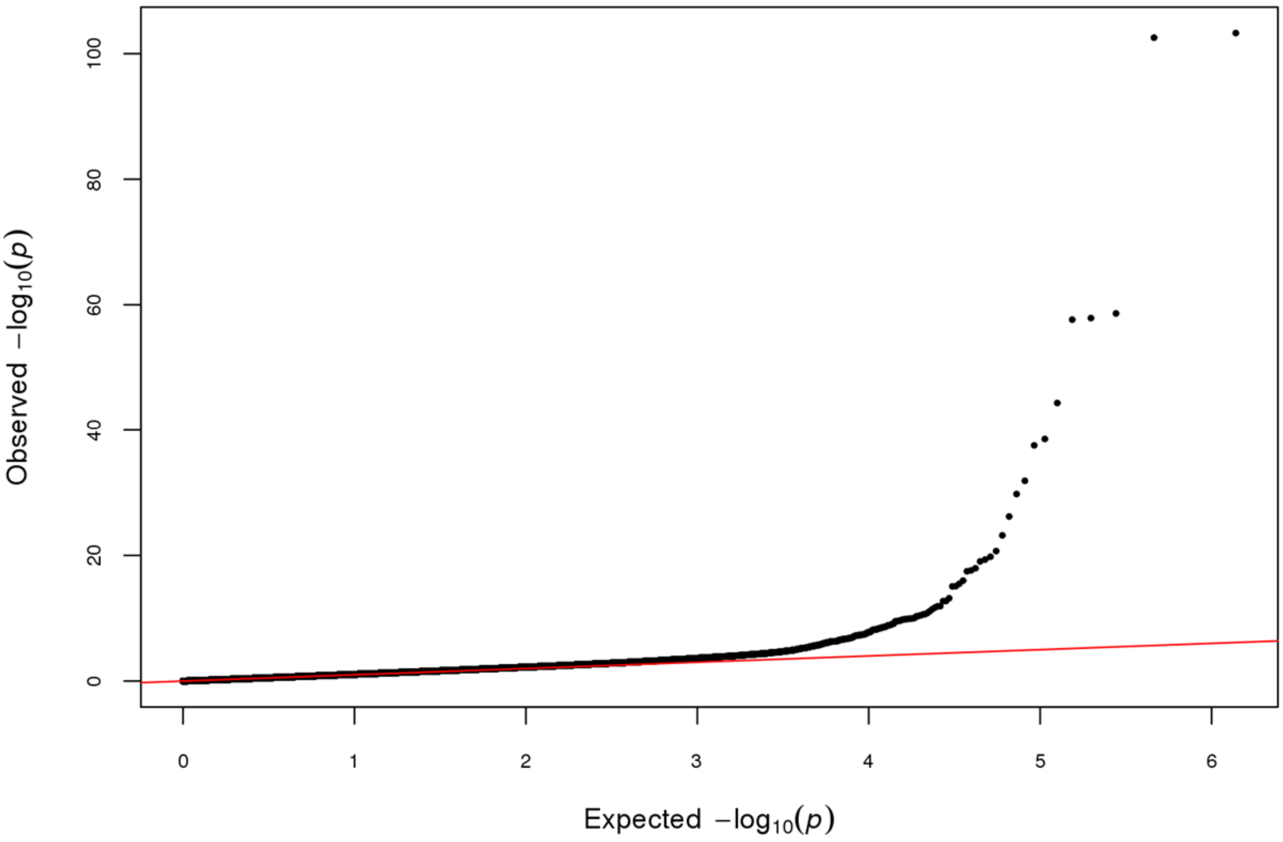

Supplementary Figure 6: Regional association plots for eyebrow thickness and monobrow in 2q12.

a) 2q12 – Eyebrow thickness

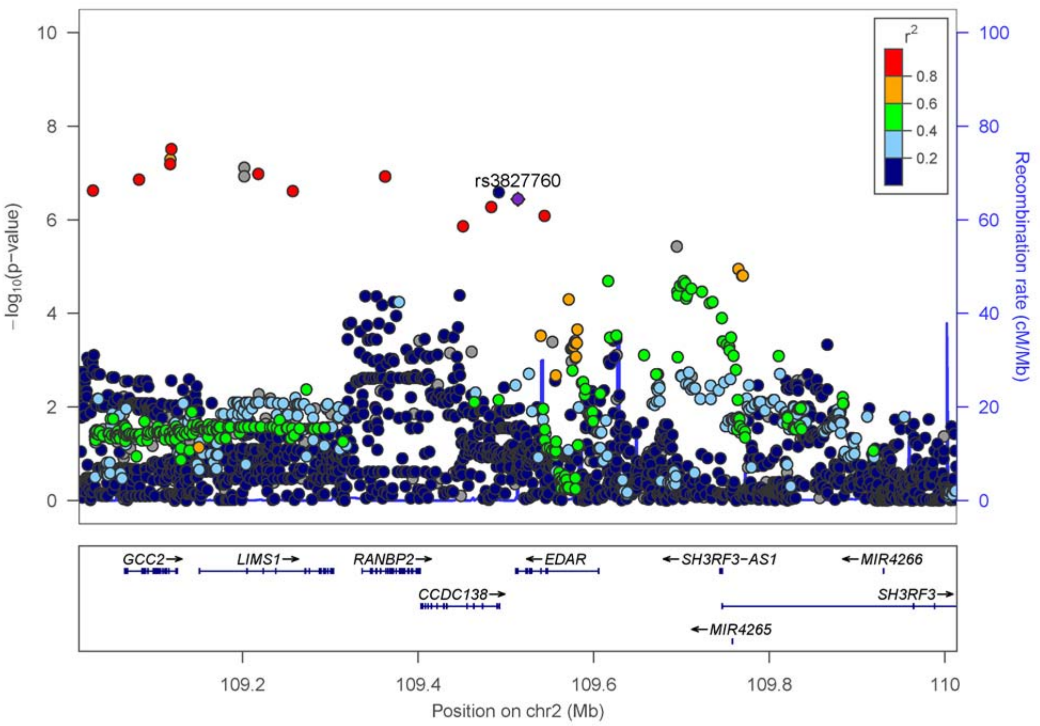

b) 2q12 – Monobrow

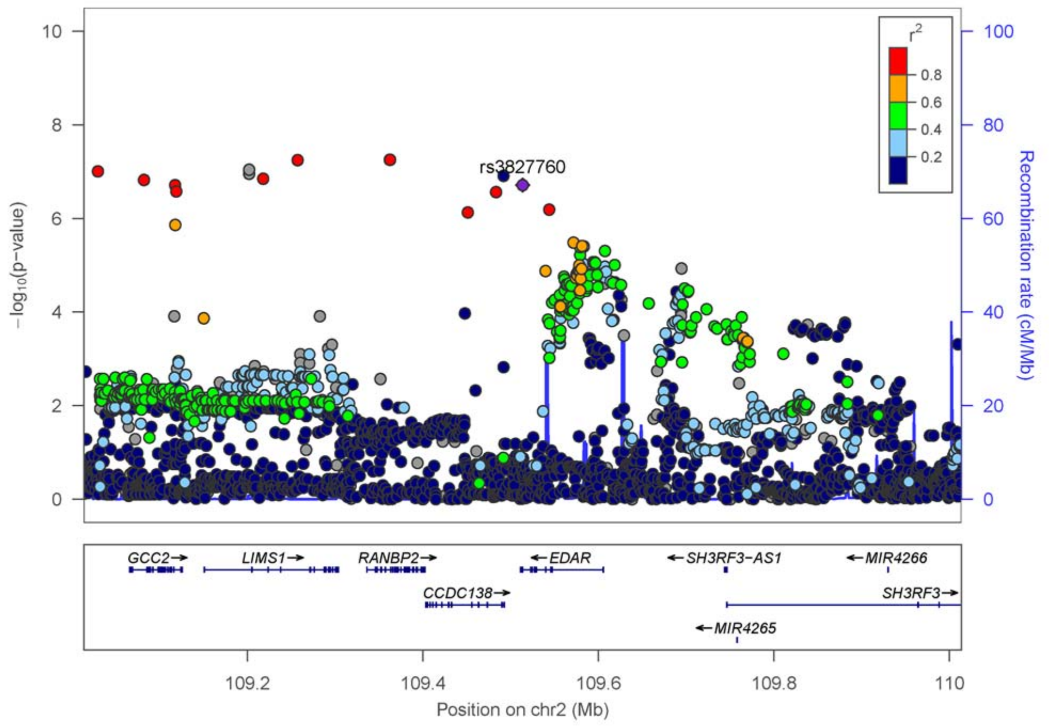

Supplementary Figure 7: The signal of association at *EDAR* intronic SNPs with beard thickness is independent of rs3827760.

Supplementary Figure 7a shows that in the GWAS for beard thickness, several SNPs in the first intron of *EDAR* have smaller *P* values than rs3827760, the missense *EDAR* index SNP strongly associated with hair shape (Table 1). These intronic SNPs are in an LD block separate from rs3827760. We evaluated whether the association signal at these SNP is independent from rs3827760 by performing tests conditioning on rs3827760 (Supplementary Figure 7b). This analysis was restricted to the 58 SNPs with *P* values $< 10^{-3}$ in the initial GWAS (panel labelled "subset" in Figure 7b), leading to a follow-up Bonferroni-corrected $-\log_{10}$(p-value) significance threshold of 3.06. Supplementary Figure 7b shows that after conditioning on rs3827760, a significant association signal is still observed for several SNPs in the first intron of EDAR, including rs365060, the index SNP associated with beard density in the initial GWAS (Table 1). For all SNPs the derived allele is associated with lower beard thickness and the direction of these effects does not change after conditioning on rs3827760 (results not shown). Supplementary Figure 7a also shows that the first intron of *EDAR* is rich in regulatory DNA elements, based on annotations from the UCSC Genome Browser (https://genome.ucsc.edu/). The bottom two panels in Supplementary Figure 7b display the CMS scores for SNPs in the EDAR region (in CEU and ASN). We observe the highly significant CMS scores in ASN corresponding to the strong signal of selection reported for variants around rs3827760, associated with hair shape (Table 1). Interestingly, we also observe significant CMS scores in CEU for the intronic EDAR variants associated here with beard thickness.

a)

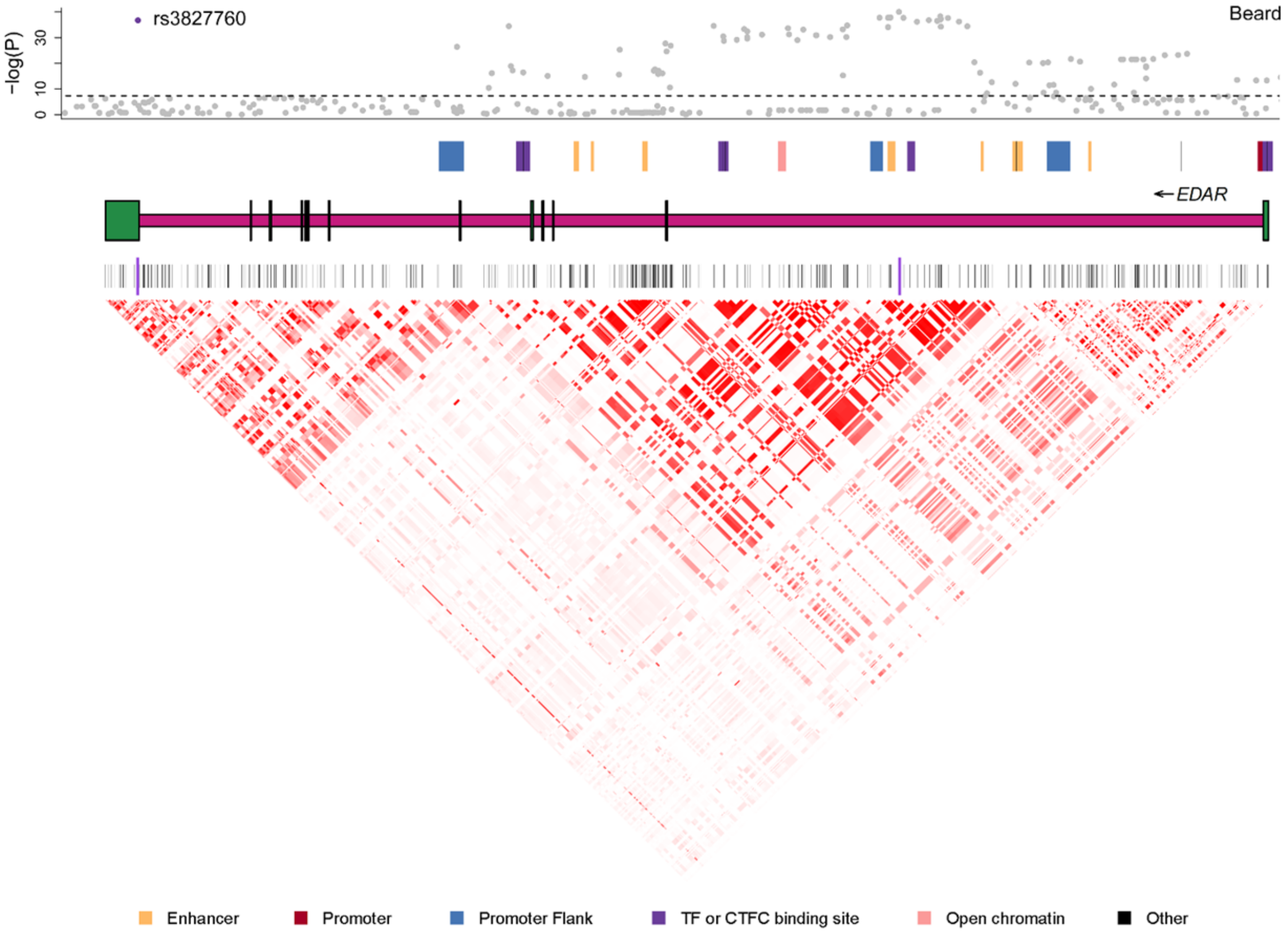

b)

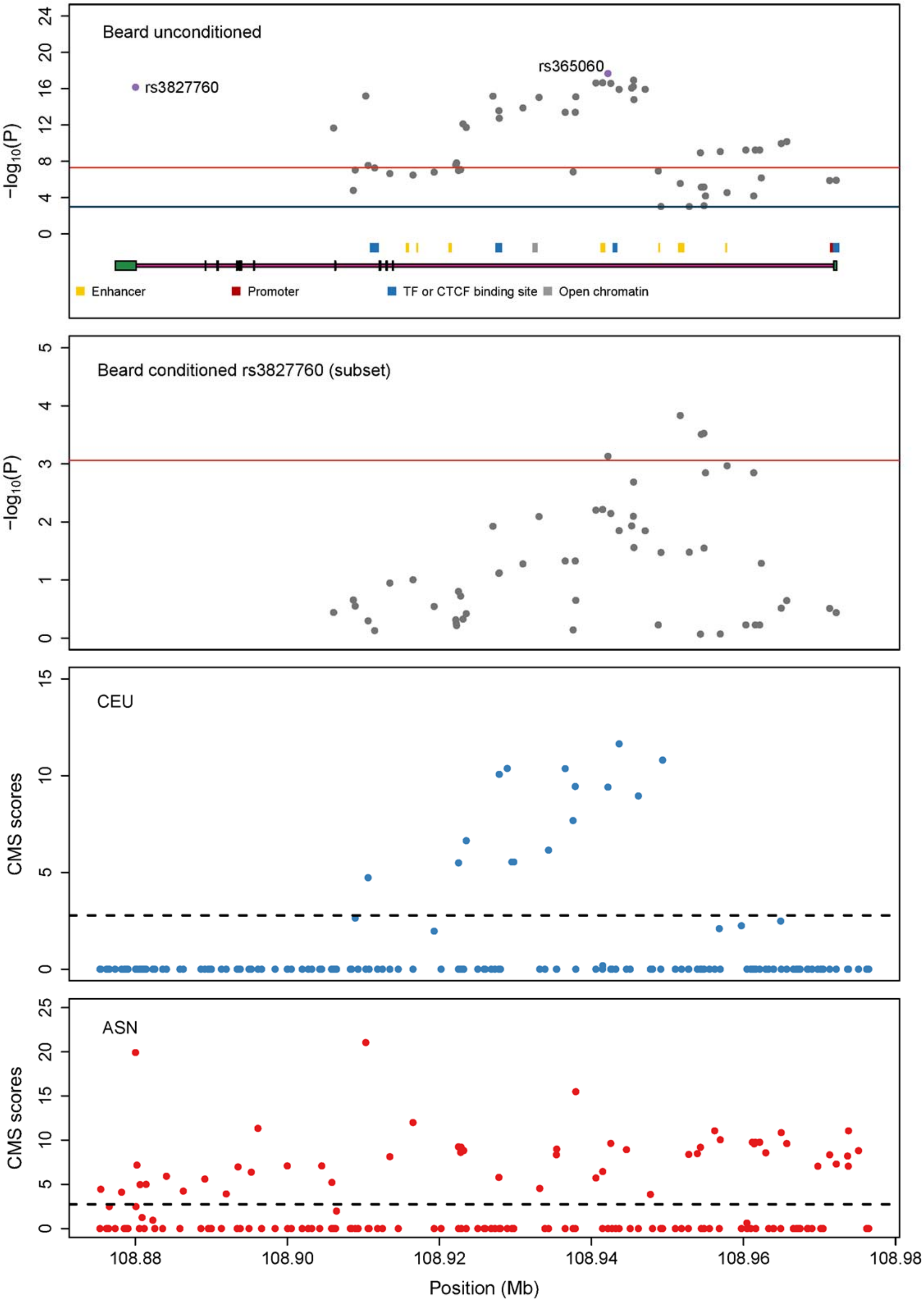

Beard unconditioned
rs3827760
rs365060
−log10(P)
Enhancer
Promoter
TF or CTCF binding site
Open chromatin
Beard conditioned rs3827760 (subset)
−log10(P)
CEU
CMS scores
ASN
CMS scores
108.88
108.90
108.92
108.94
108.96
108.98
Position (Mb)

Supplementary Figure 8: Regional association plots for regions not shown in Figure 3

The index SNP in each region (Table 1) is shown as a purple diamond. At the top of the figure are shown the association results (on a -log10 *P* scale; left y-axis) for all genotyped and imputed SNPs. The dot color indicates the strength of LD (r2) between the index SNP and each SNP (based on the 1000genomes AMR dataset). Recombination rate across the region, in the AMR data, is shown as a continuous blue line (scale on the right y-axis). Genes in the region are shown in the middle. Plots for regions representing novel associations include at the bottom an LD heatmap similar to those shown in Figure 3 (using D', red indicating D'=1 and white indicating D'=0). Plots for regions that have been previously associated with the same trait omit the LD heatmap.

a) 1q21 – Hair Shape

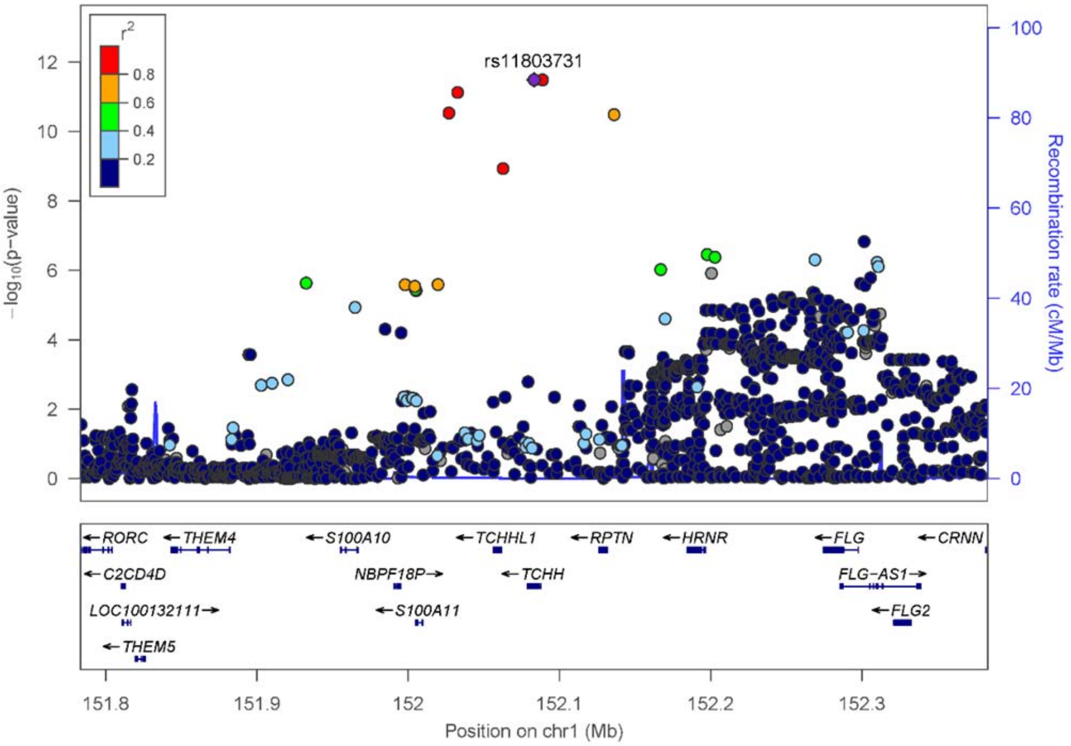

b) 10p14 – Hair shape

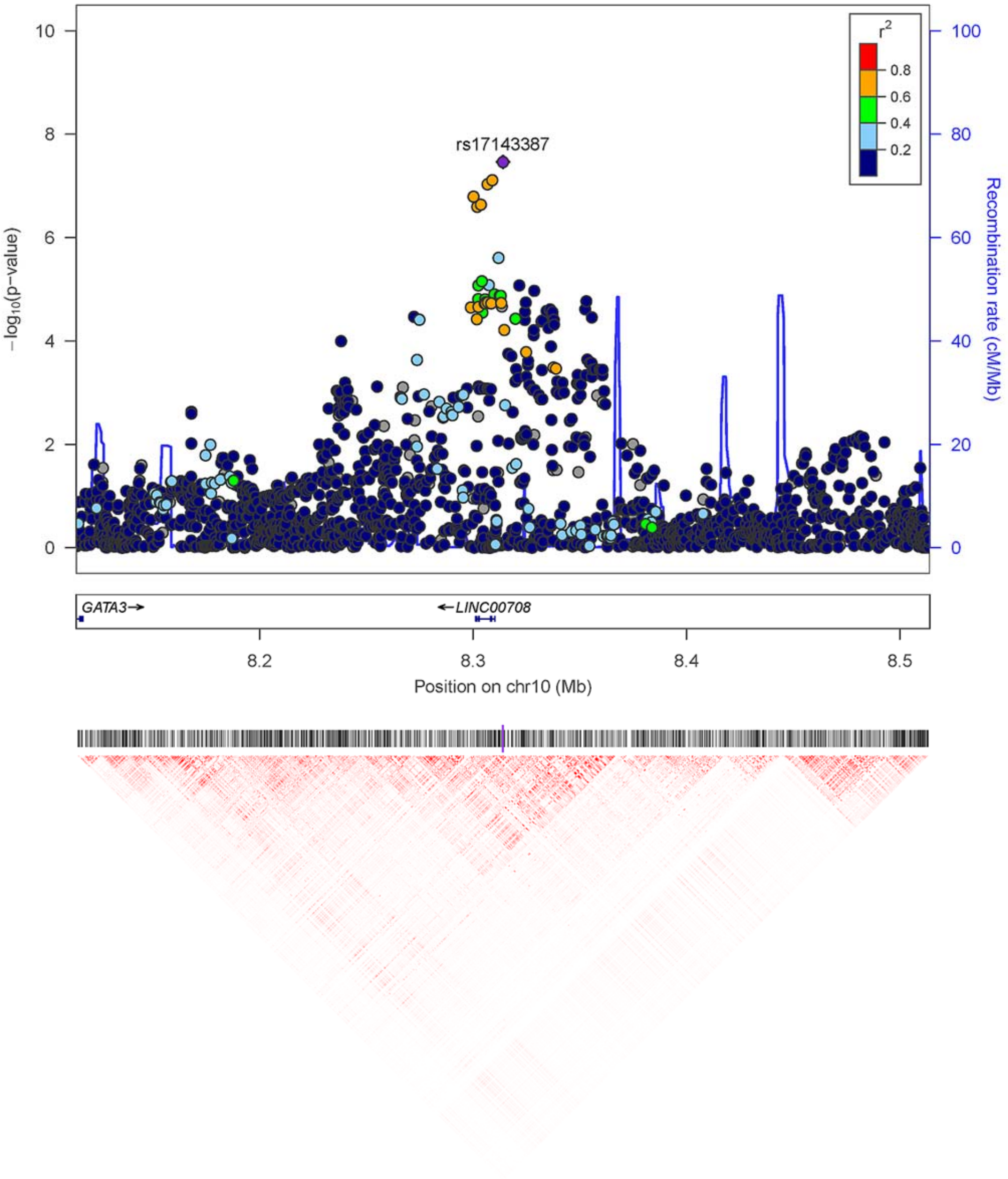

c) 4q12 – Beard

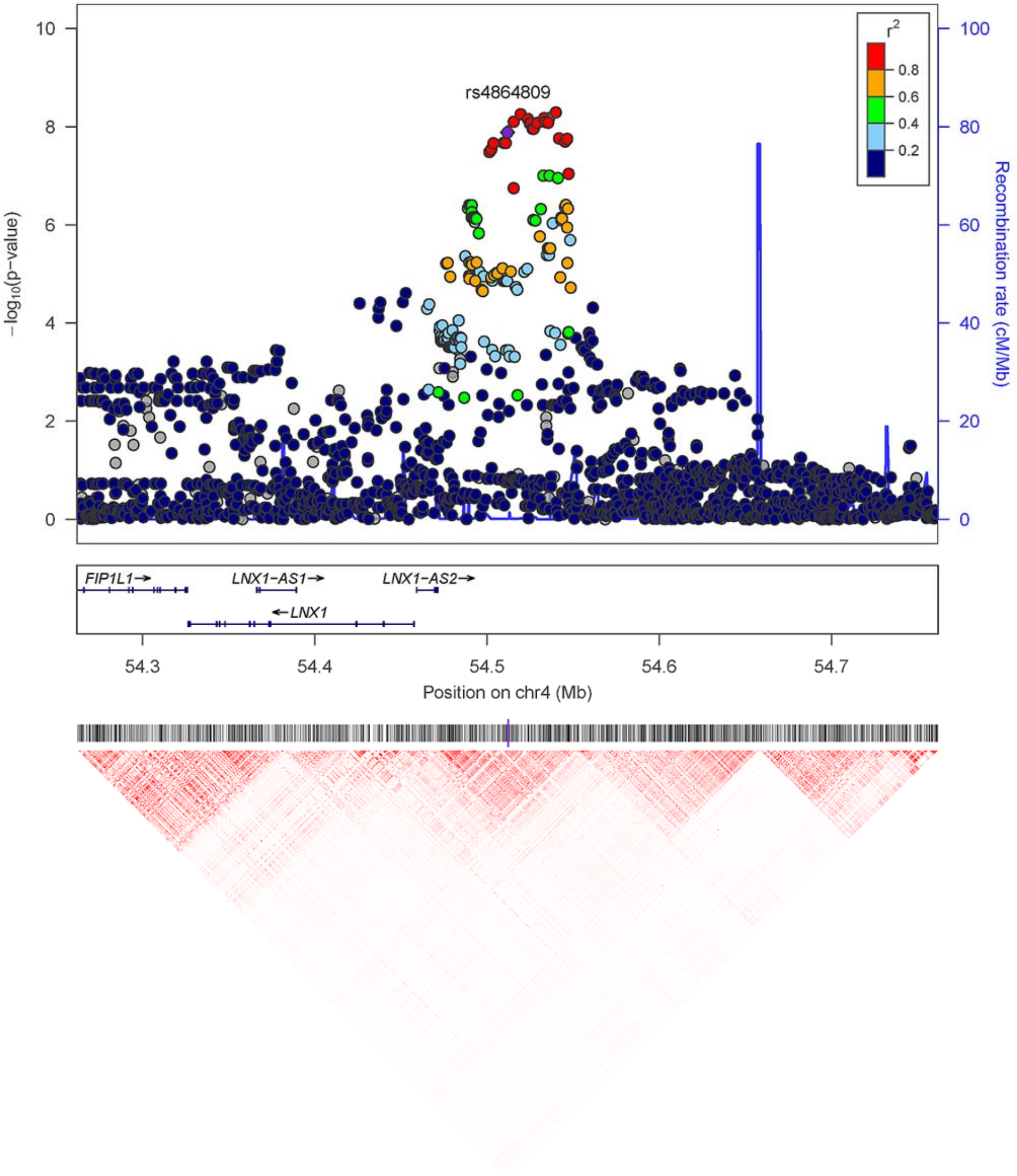

rs4864809
r2
0.8
0.6
0.4
0.2
10
8
6
4
2
0
100
80
60
40
20
0
−log10(p−value)
Recombination rate (cM/Mb)
FIP1L1→
LNX1−AS1→
LNX1−AS2→
←LNX1
54.3
54.4
54.5
54.6
54.7
Position on chr4 (Mb)

d) 6q21 - Beard

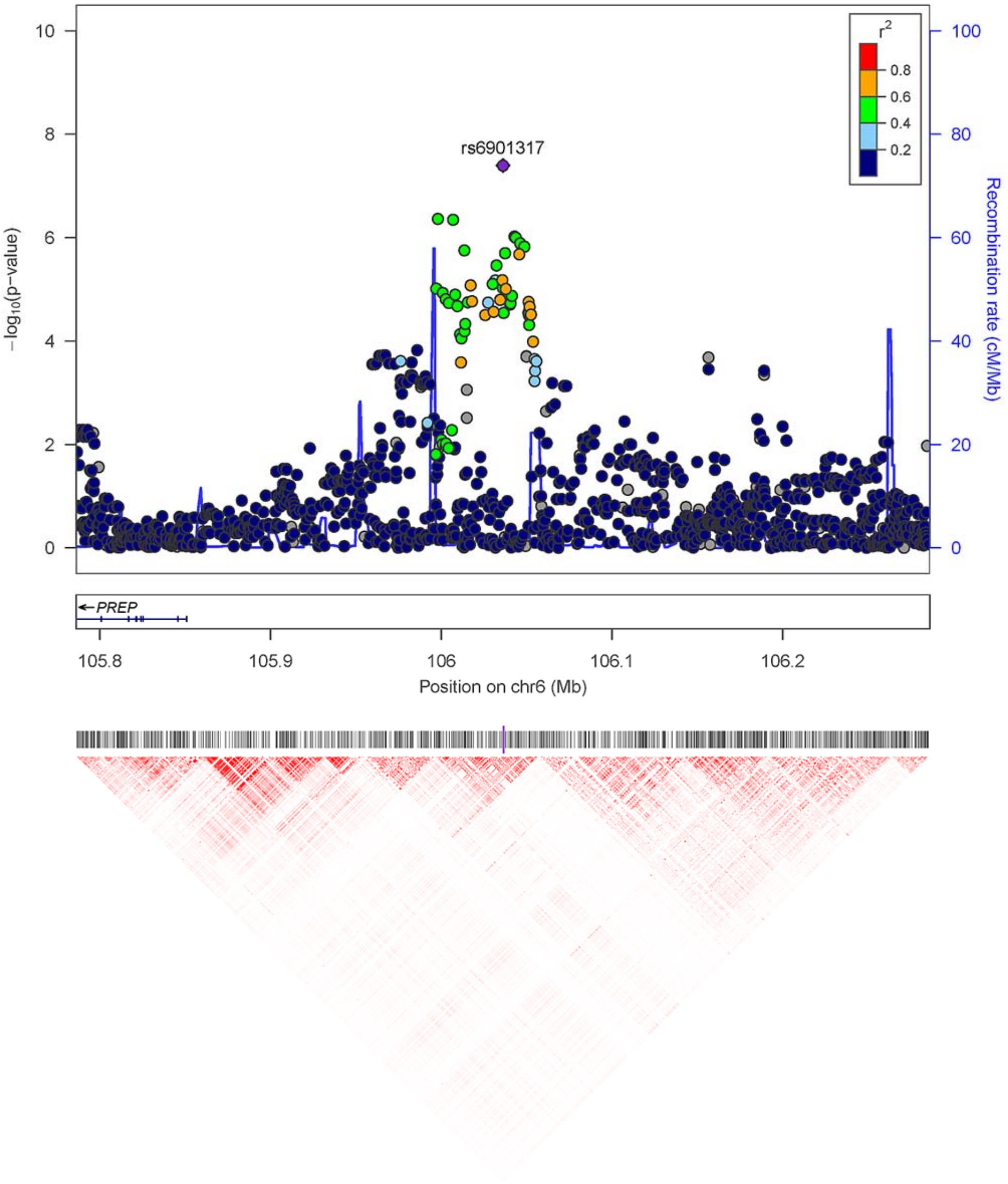

rs6901317
r²
0.8
0.6
0.4
0.2
10
8
6
4
2
0
100
80
60
40
20
0
−log10(p−value)
Recombination rate (cM/Mb)
PREP
105.8
105.9
106
106.1
106.2
Position on chr6 (Mb)

e) 7q31 - Beard

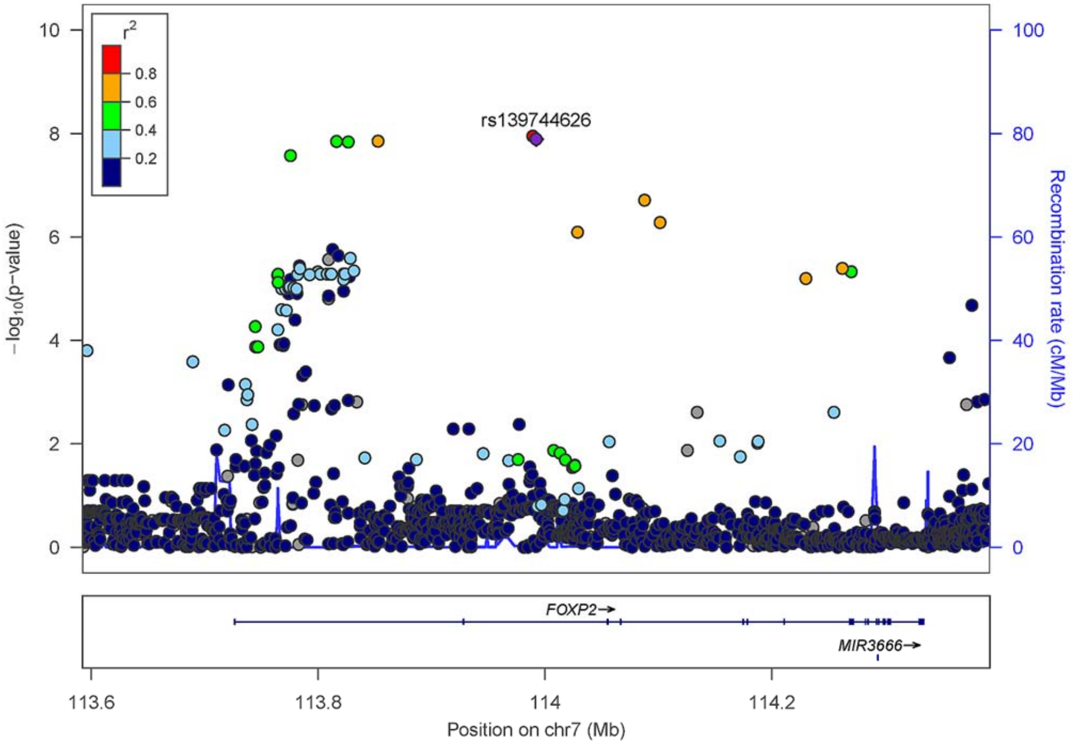

r²
0.8
0.6
0.4
0.2
rs139744626
10
8
6
4
2
0
−log10(p−value)
100
80
60
40
20
0
Recombination rate (cM/Mb)
FOXP2→
MIR3666→
113.6
113.8
114
114.2
Position on chr7 (Mb)

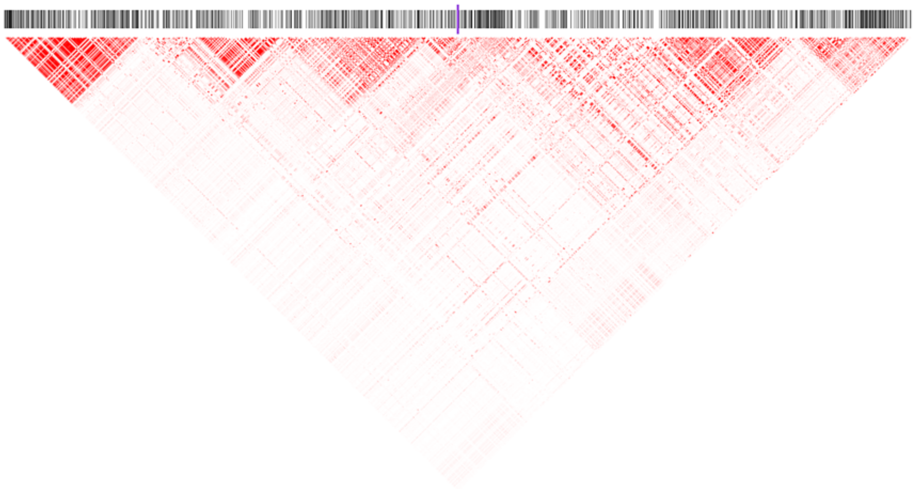

f) 5p13 – Hair Color

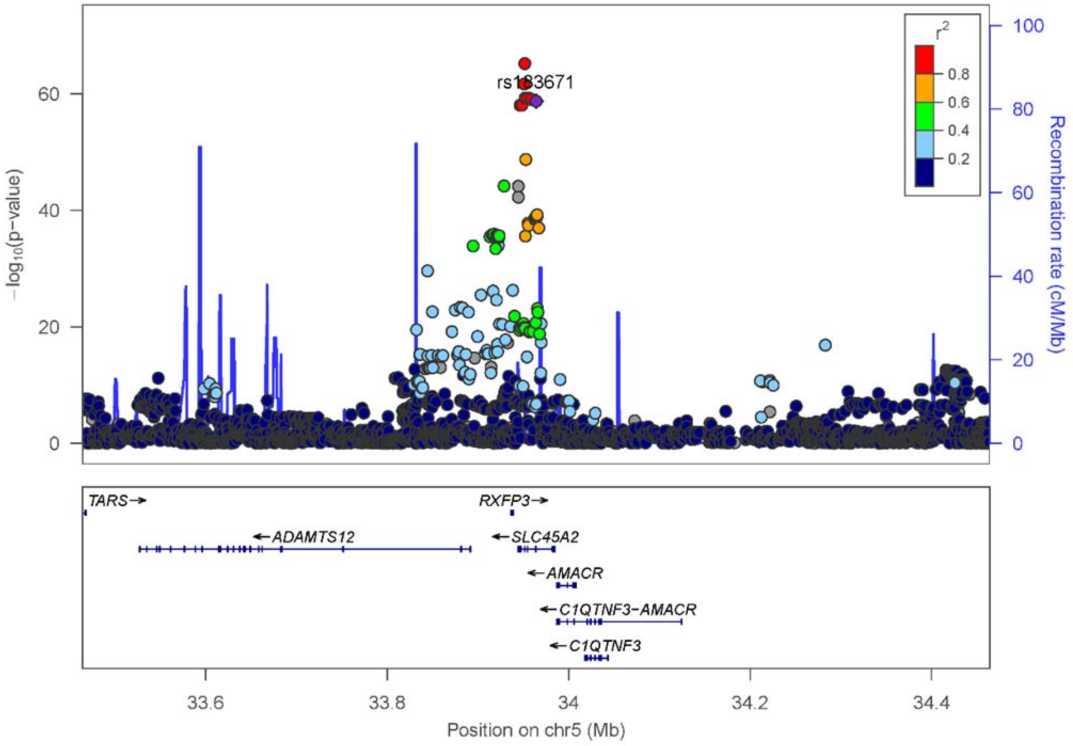

g) 6p25 – Hair Color

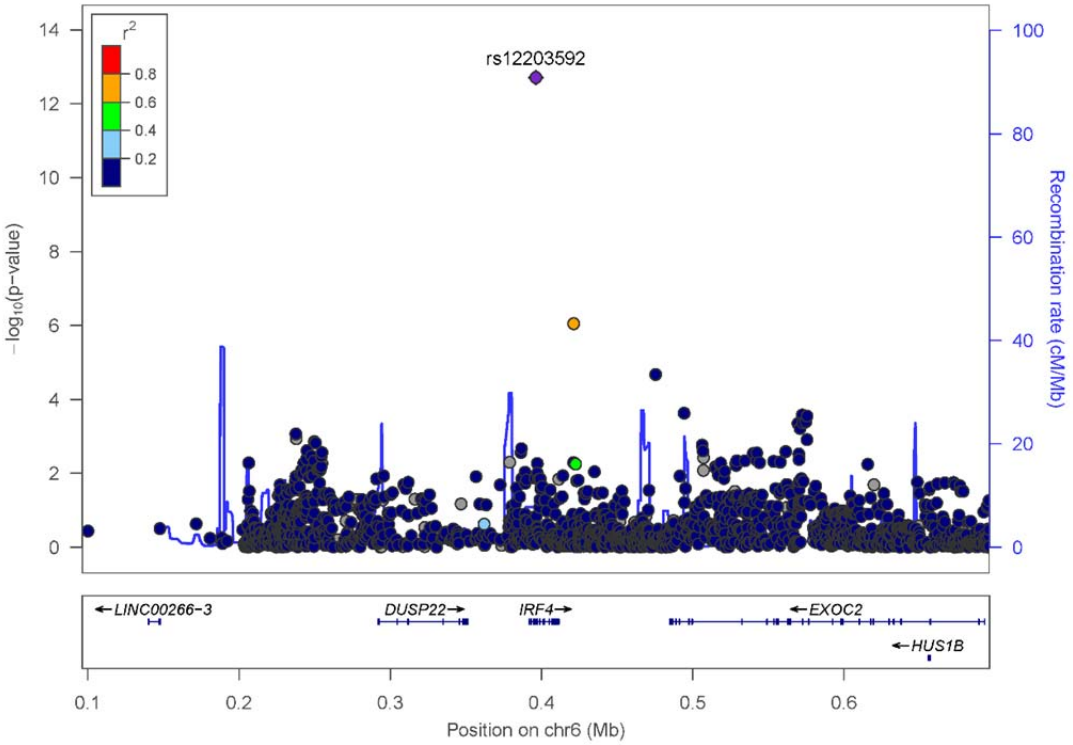

h) 11q14 – Hair Color

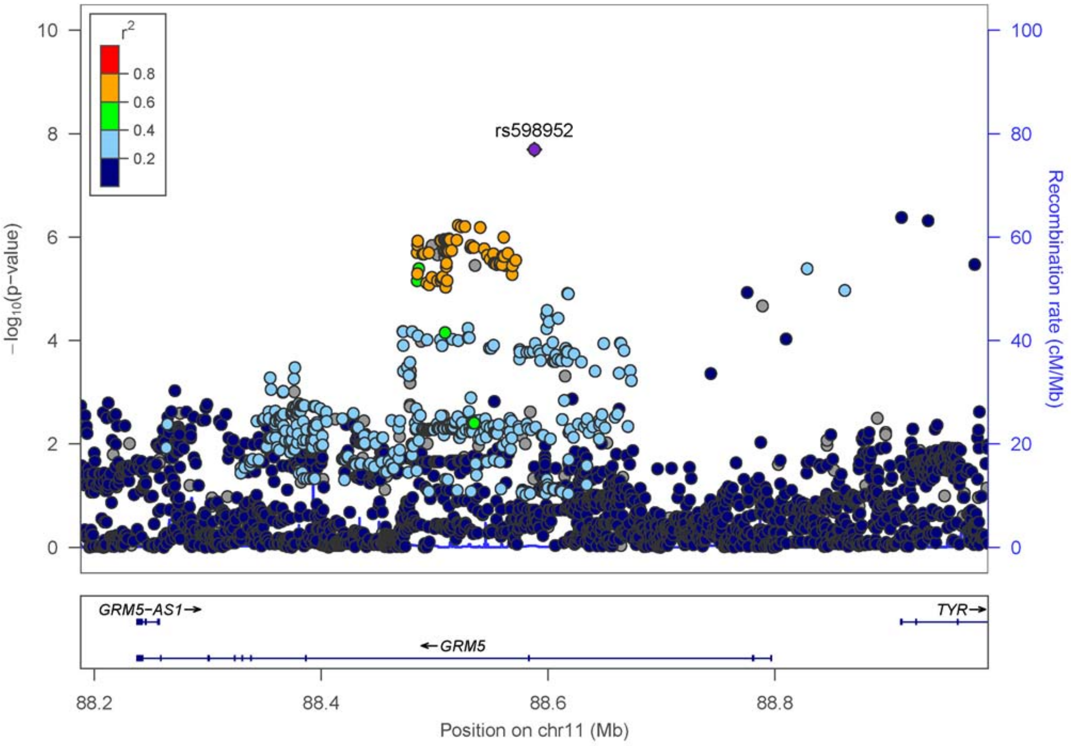

i) 15q13 – Hair Color

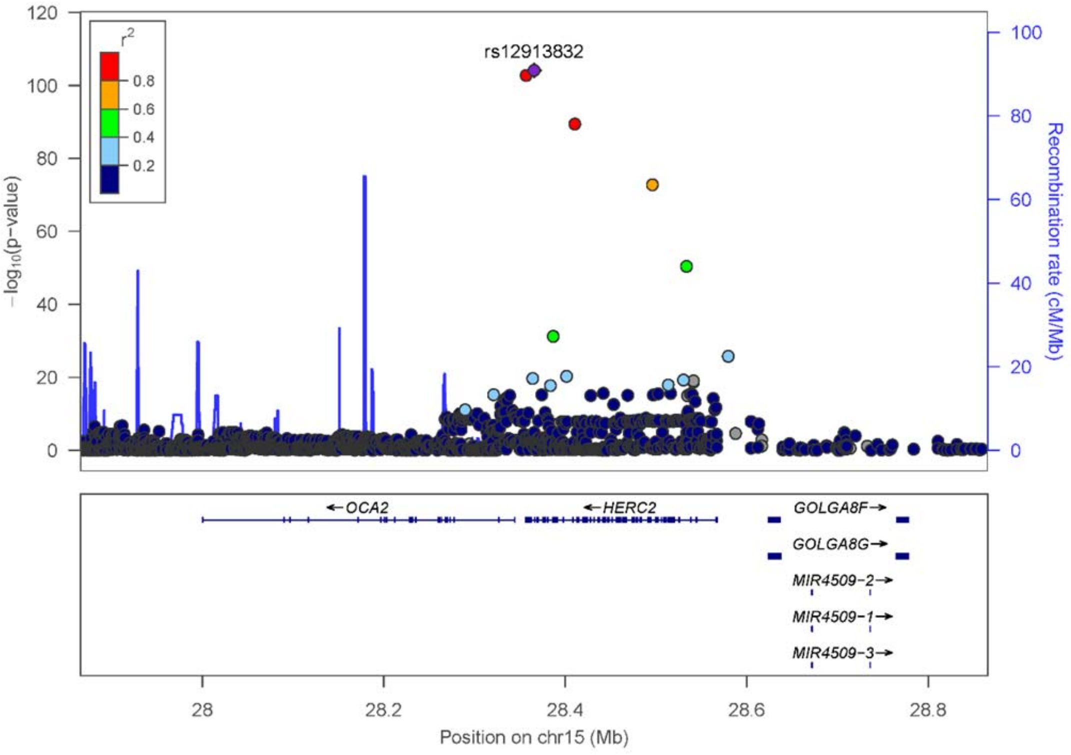

j) 15q21 – Hair Color

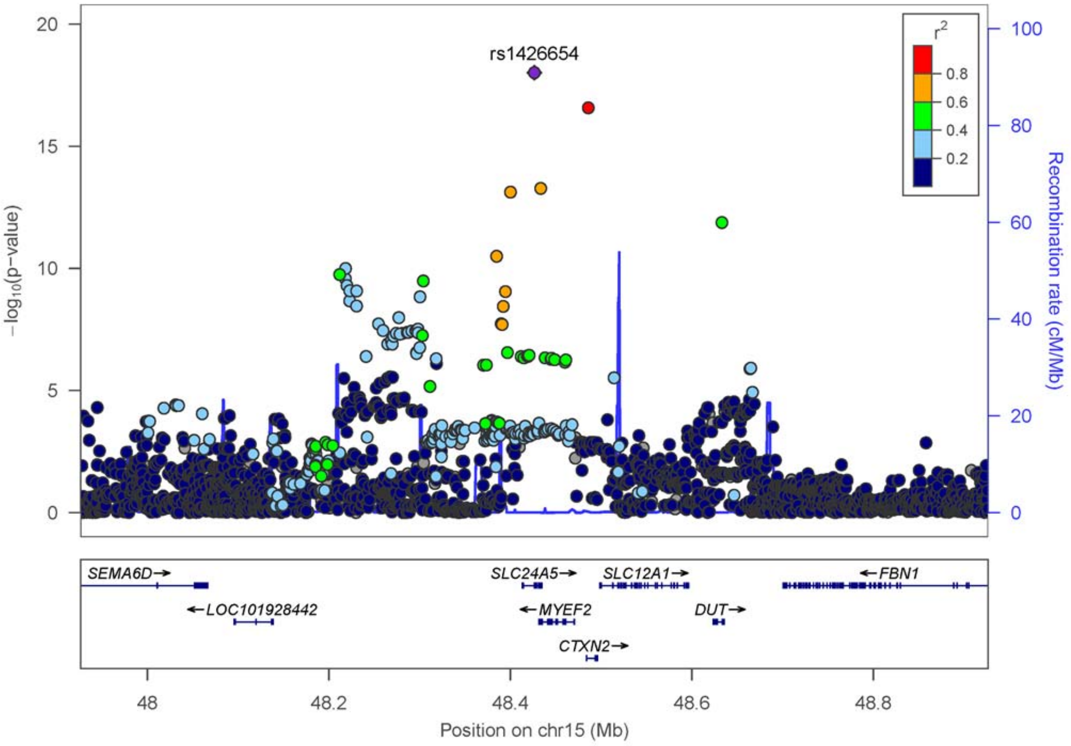

k) 10q22 - Balding

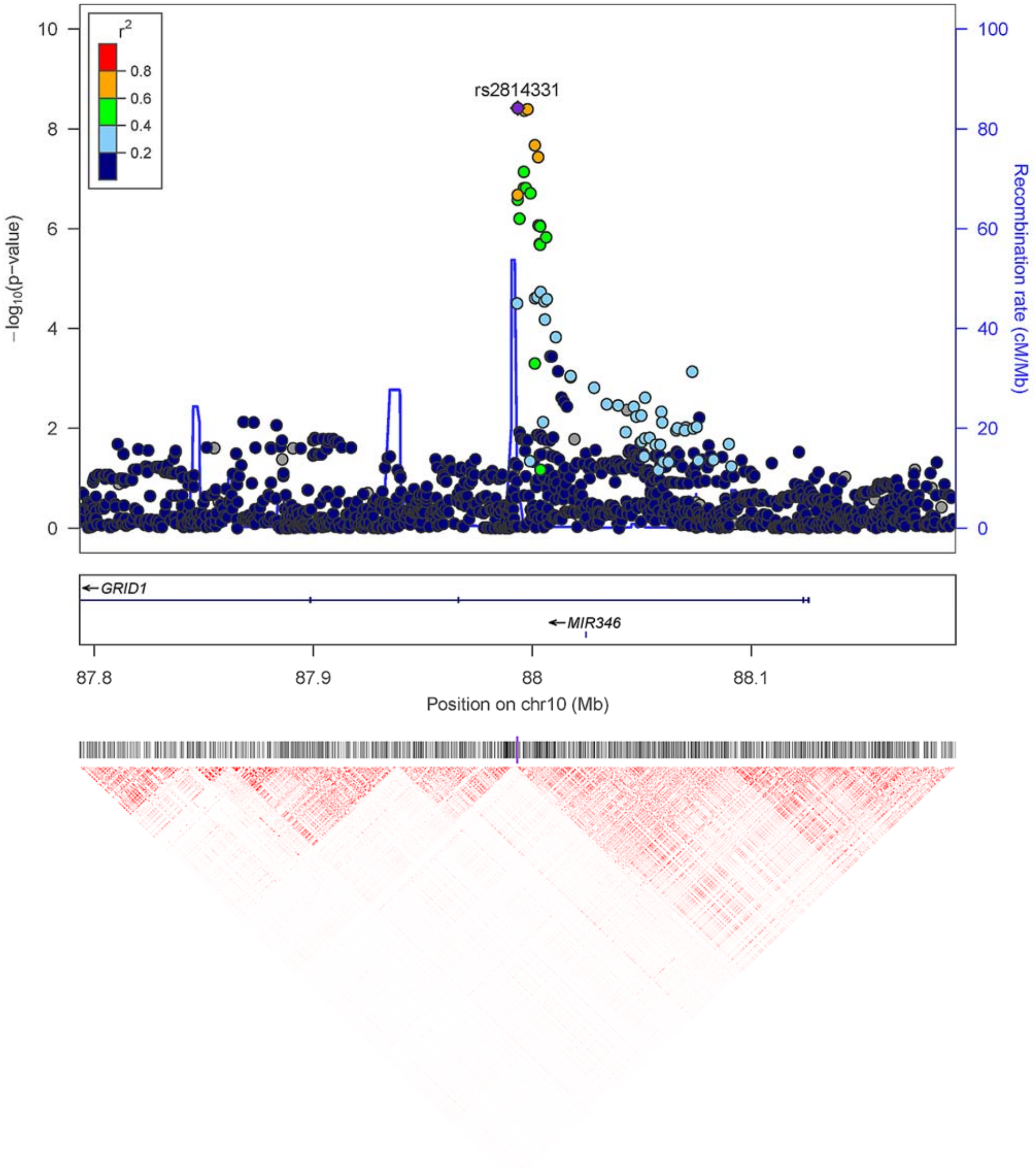

l) Xq12 – Balding

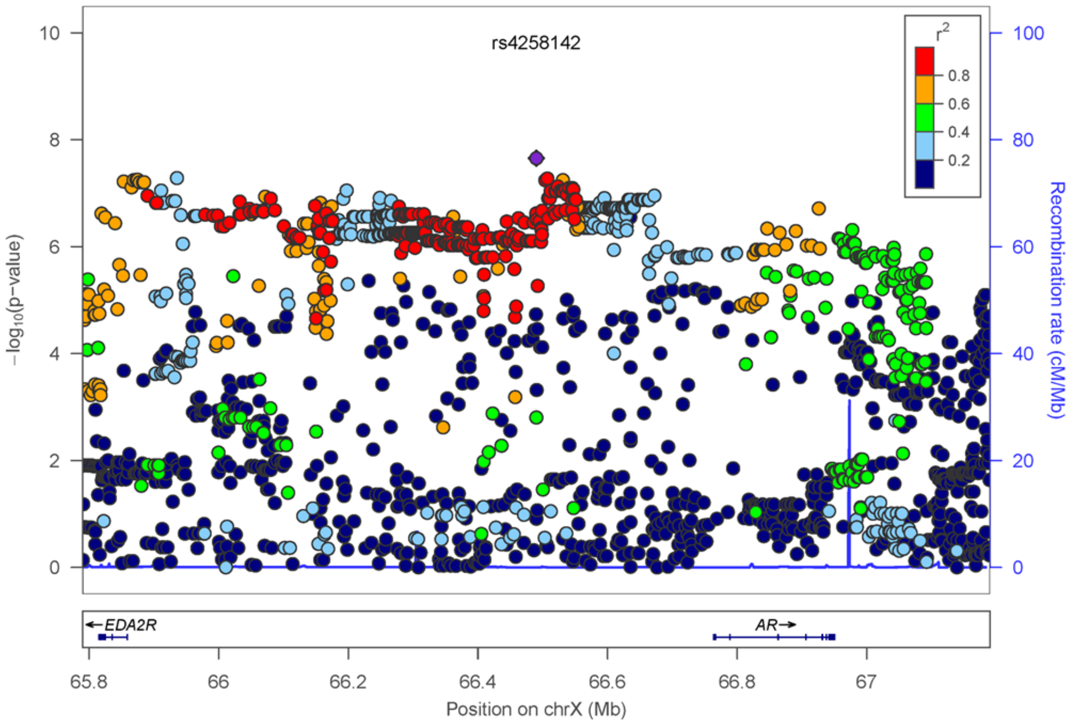

Supplementary Figure 9: Proprotein convertase cleavage site in PRSS53 (Q30R).

A)

Analysis of the complete amino acid sequence of PRSS53 and PRSS53 Q30R was performed at http://elm.eu.org/. No proprotein corvertase sites were detected in PRSS53. Potential proprotein convertase sites detected in PRSS53 Q30R are boxed.

PRSS53:

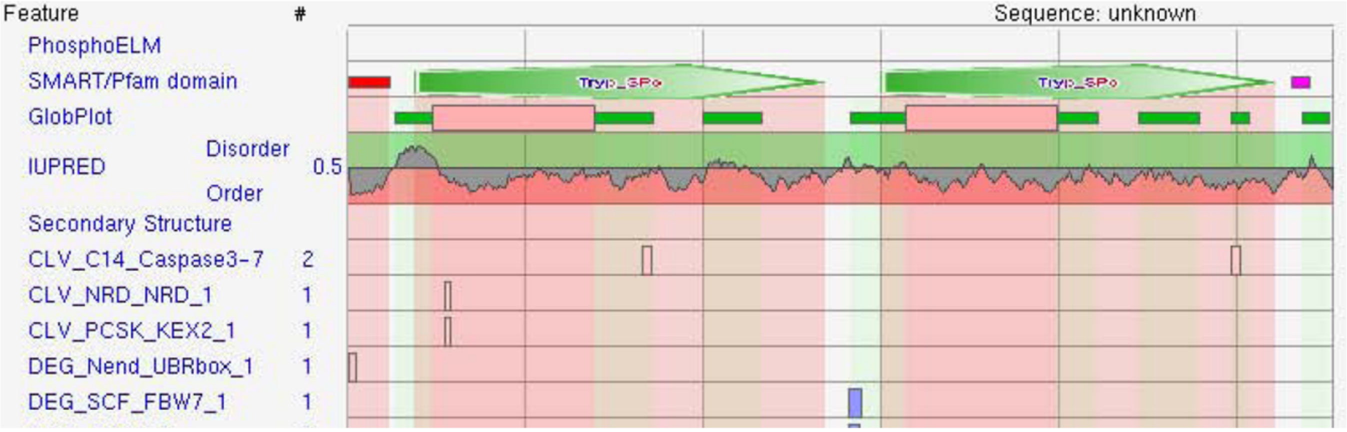

PRSS53 Q30R:

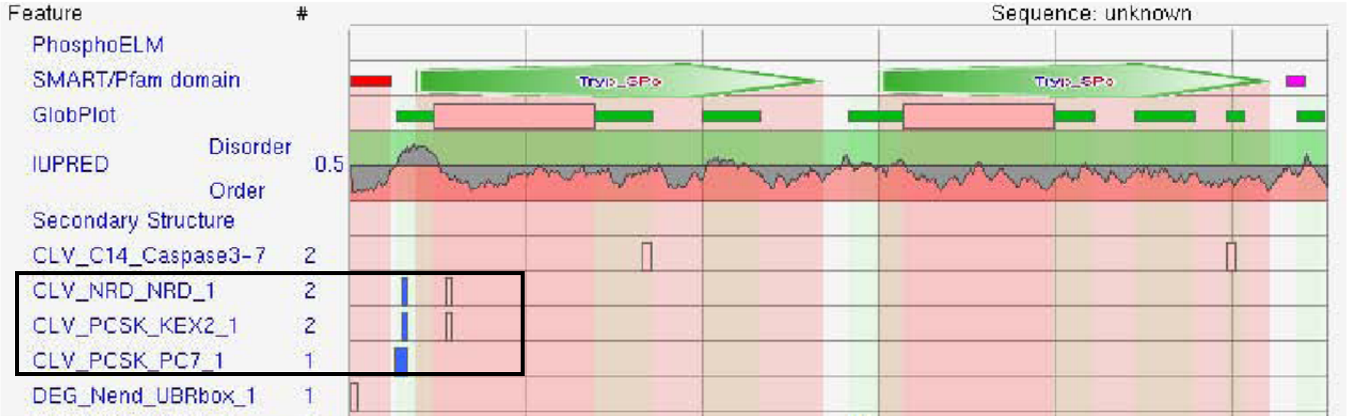

B)

At the top are shown Sanger sequence profiles for the *PRSS53* gene region encoding the Q30R substitution associated with hair shape. Below is shown the N-terminal sequence of PRSS53 Q30R with the location of the signal peptide cleavage site indicated by blue arrows. In the case of PRSS53 30R (on the right), the putative proprotein convertase cleavage site is highlighted by another blue arrow.

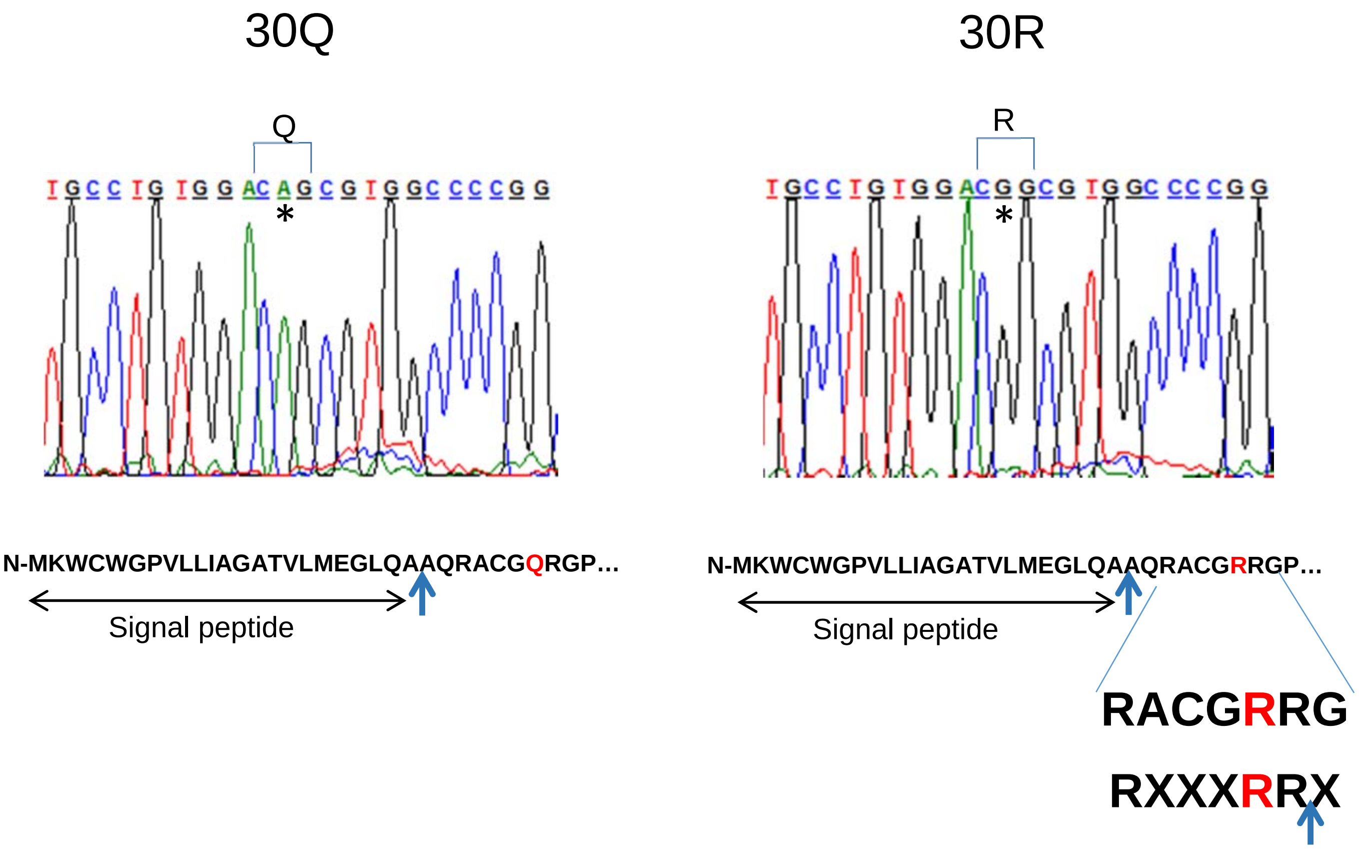

Supplementary Figure 10: Distribution of balding trait across sexes

As indicated in Supplementary Figure 1, Balding was coded into three categories: none, medium and high. The frequency of balding for males and females separately and in the full sample is displayed below.

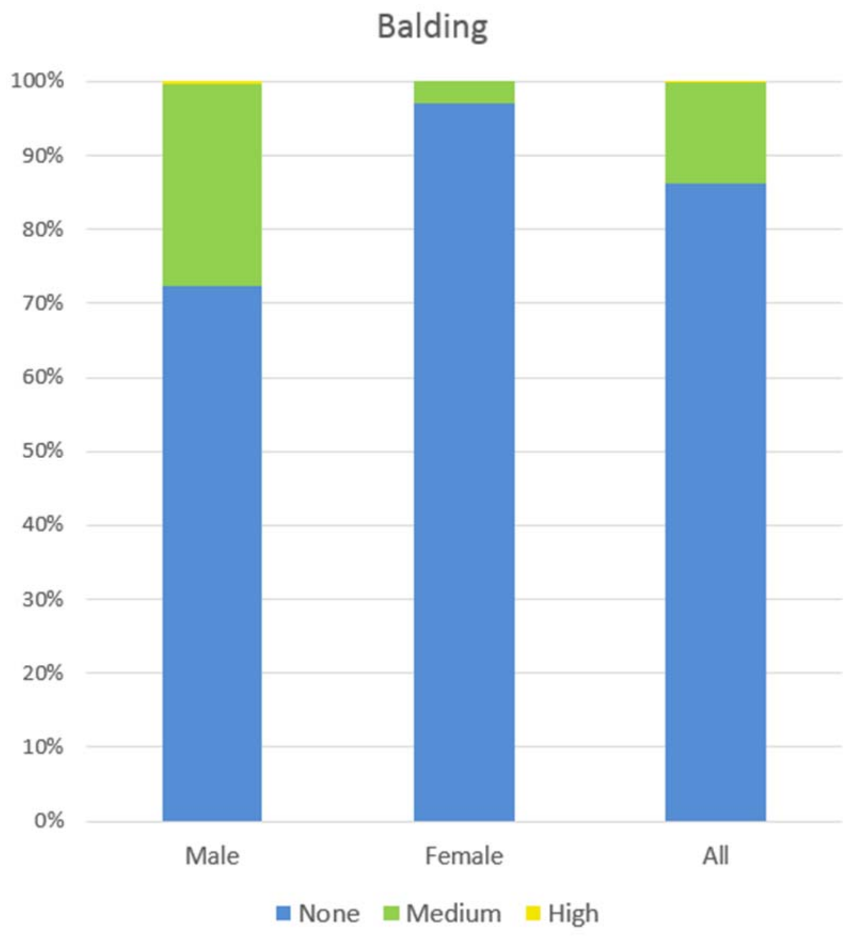

Supplementary Figure 11: Expression of PRSS53 in 293-EBNA cells

Comparison of PRSS53 and PRSS53 (Q30R) from cell extracts (A) and media (B), after expression in 293-EBNA cells cultured in the absence (-) or presence (+) of DECA (decanoyl-RVKR-CMK, a pro-protein convertase inhibitor). These are uncropped western blot images corresponding to those shown in Figure 5 a-b and are labelled below as in Figure 5.

Figure 5a:

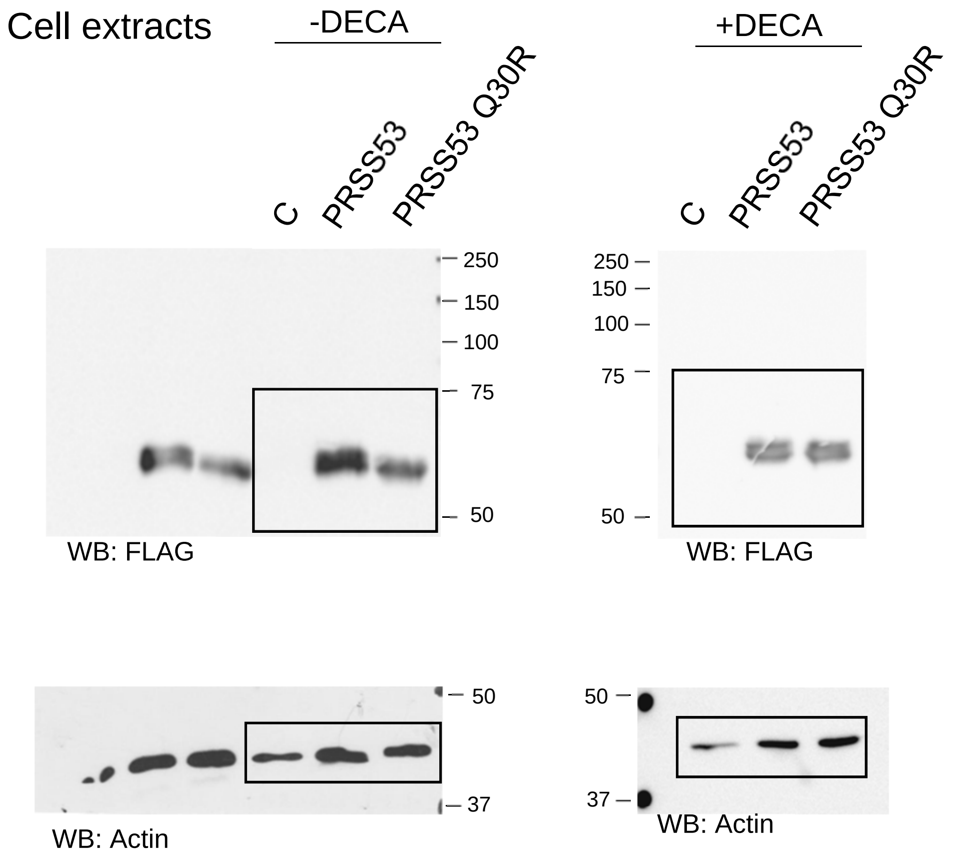

Figure 5b:

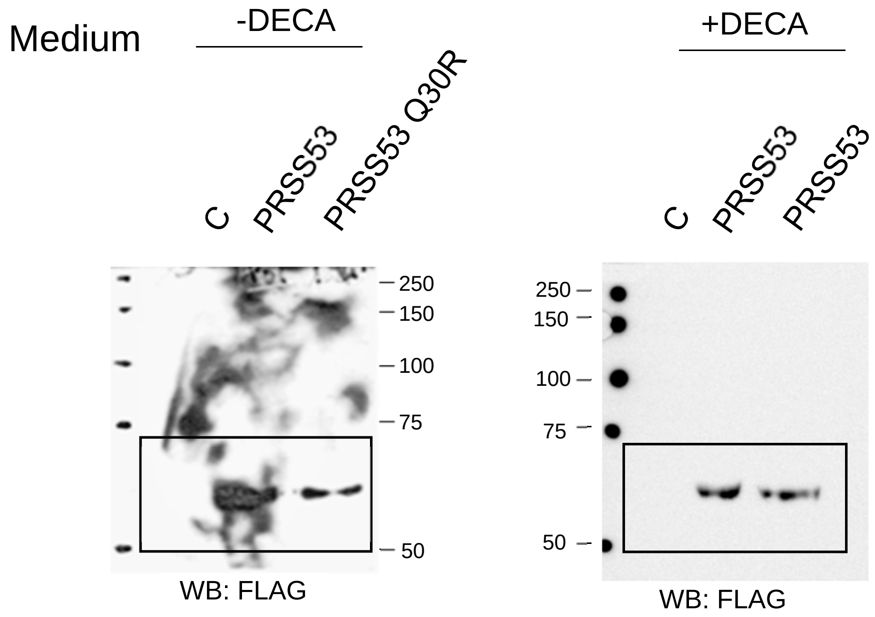

SUPPLEMENTARY TABLES

Supplementary Table 1: Features of the CANDELA individuals included in this study.

	Total	Colombia	Brazil	Chile	Mexico	Peru
**Sample size**	6357	1507	651	1745	1207	1247
**Percentage**	100	23.7	10.2	27.5	19.0	19.6
**% Female**	54.0	55.9	68.5	39.6	60.3	58.4
**Age (years)**						
Min	18	18	18	18	18	18
Mean	24.2	24.0	25.8	25.2	24.4	22.2
Max	45	40	45	45	44	44
S.D.	5.7	5.3	6.3	5.8	5.6	5.2
**Age, for Males**						
Min	18	18	18	18	18	18
Mean	24.9	24.7	25.8	25.3	25.1	23.0
Max	45	40	45	45	44	44
S.D.	5.7	5.5	6.4	5.5	5.6	5.7
**Age, for Females**						
Min	18	18	18	18	18	18
Mean	23.8	23.5	25.4	25.2	24.0	21.6
Max	45	40	44	45	41	42
S.D.	5.7	5.0	4.2	6.2	4.7	4.7

Supplementary Table 2: Correlation of hair traits and covariates in the CANDELA sample included in the GWAS (N=6,357)

A) Correlation between hair traits:

Pearson correlation coefficient values are presented in the lower left triangle while corresponding permutation *P* values are presented in the upper right triangle. Correlations with significant *P* values (<0.001, Bonferroni-adjusted threshold) and their corresponding *P* values are highlighted in bold.

	**HC**	**HG**	**HS**	**BL**	**BT**	**ET**	**MB**
**Hair color (HC)**		3.9E-03	**8.6E-07**	**1.7E-04**	1.5E-02	**8.3E-06**	2.1E-02
**Hair graying (HG)**	-0.03		1.9E-01	**3.5E-52**	**9.1E-11**	**6.6E-06**	3.8E-01
**Hair shape (HS)**	**0.05**	0.01		7.5E-01	4.4E-03	2.1E-01	6.5E-03
**Balding (BL)**	**0.04**	**0.16**	0.00		**4.3E-14**	2.2E-03	**1.2E-09**
**Beard thickness (BT)**	-0.05	**0.13**	0.06	**0.15**		**5.7E-20**	**1.3E-12**
**Eyebrow thickness (ET)**	**0.09**	**-0.09**	-0.03	-0.06	**0.18**		**2.0E-32**
**Monobrow (MB)**	0.03	-0.01	-0.04	**0.09**	**0.14**	**0.24**	

B) Correlation between hair traits and covariates:

Pearson correlation coefficient

Trait	Sex	Age	Ancestry		
			African	European	Native American
Hair color	**-0.10**	-0.02	0.01	**-0.32**	**0.31**
Hair graying	**-0.05**	**0.56**	0.00	0.03	-0.03
Hair shape	0.02	-0.01	**0.15**	**0.11**	**-0.16**
Balding	**-0.35**	**0.15**	**-0.05**	**-0.07**	**0.08**
Beard thickness	**-**	**0.28**	0.04	**0.30**	**-0.30**
Eyebrow thickness	**-**	**-0.17**	0.03	0.05	-0.06
Monobrow	-	0.02	0.00	**0.11**	**-0.10**

***P* value**

Trait	Sex	Age	Ancestry		
			African	European	Native American
Hair color	**1.3E-16**	1.2E-01	5.4E-01	**1.5E-10**	**1.0E-144**
Hair graying	**1.5E-05**	**0.0E+00**	8.9E-01	6.7E-03	7.8E-03
Hair shape	1.9E-01	6.8E-01	**4.6E-36**	**2.2E-20**	**8.1E-37**
Balding	**5.5E-190**	**3.3E-36**	**1.0E-04**	**8.3E-08**	**1.3E-10**
Beard thickness	**-**	**1.6E-45**	5.6E-02	**1.4E-51**	**7.3E-53**
Eyebrow thickness	**-**	**4.2E-17**	1.6E-01	9.6E-03	3.1E-03
Monobrow	-	1.6E-01	9.9E-01	**7.8E-13**	**3.5E-12**

Individual continental ancestry was estimated from the chip data obtained here (Supplementary Figure 2).

Sex was coded as: female=1, male=0.

Correlations with significant *P* values (<0.001, Bonferroni-adjusted threshold), are highlighted in bold.

Supplementary Table 3: Narrow-sense heritability (h^2) of hair traits, estimated from the population data obtained here.

**Trait**	h^2	**S.E.**	**P-value**
Hair color	1.00	0.05	0.0E+00
Hair graying	0.27	0.05	4.4E-13
Hair shape	0.64	0.05	0.0E+00
Balding	0.39	0.05	0.0E+00
Beard thickness	0.74	0.12	0.0E+00
Eyebrow thickness	0.37	0.11	7.2E-10
Monobrow	0.74	0.12	0.0E+00

Supplementary Table 4: Meta-analysis association *P* values for index SNPs.

For the index SNPs in Table 1 we performed association tests independently in the five country samples and the *P* values combined using a meta-analysis. For each SNP we provide below the basic meta-analysis *P* value, a test of heterogeneity *P* value (obtained through Cochran's *Q* statistic) and a random effects (R.E. model) meta-analysis *P* value. With 20 tests, the Bonferroni-corrected significance threshold for P-values is 0.05/20 = 2.5E-03. Both meta-analysis *P* values are significant at this level for all SNPs. For 4 SNPs there is significant evidence of heterogeneity, based on the Cochran's *Q P* value (highlighted in bold). For those SNPs one should preferably consider the random effects *P* value, while for the other SNPs one should consider the basic meta-analysis P-value.

**Chromosome**	**SNP**	**Trait**	***P* value**	***Cochran's Q P* value**	***P* value (R.E. model)**
1	rs11803731	Hair Shape	1.94E-12	1.42E-01	1.34E-07
2	rs3827760	Hair Shape	2.11E-131	**1.00E-04**	3.91E-12
10	rs17143387	Hair Shape	4.73E-08	7.78E-01	4.73E-08
16	rs11150606	Hair Shape	2.22E-09	8.95E-01	2.22E-09
5	rs183671	Hair Color	2.74E-59	**1.00E-04**	2.18E-07
6	rs12203592	Hair Color	6.42E-10	6.10E-02	1.89E-05
11	rs598952	Hair Color	2.60E-11	3.43E-02	4.03E-05
15	rs12913832	Hair Color	1.59E-88	**1.00E-04**	4.26E-09
15	rs1426654	Hair Color	1.63E-23	**1.00E-04**	1.11E-04
6	rs12203592	Hair Graying	1.66E-12	5.76E-02	4.25E-06
2	rs365060	Beard Thickness	3.34E-17	1.67E-01	7.36E-09
4	rs4864809	Beard Thickness	2.27E-07	9.55E-01	2.27E-07
6	rs6901317	Beard Thickness	2.07E-07	6.63E-02	1.16E-03
7	rs117717824	Beard Thickness	9.94E-08	3.74E-01	2.71E-07
2	rs3827760	Eyebrow Thickness	3.95E-04	2.80E-01	1.74E-03
3	rs112458845	Eyebrow Thickness	1.11E-10	5.90E-02	5.59E-04
2	rs3827760	Monobrow	6.01E-07	4.53E-01	6.01E-07
2	rs2218065	Monobrow	1.77E-07	7.79E-02	3.40E-04
10	rs2814331	Balding	1.95E-10	2.16E-01	7.00E-07
X	rs4258142	Balding	2.48E-04	4.20E-01	2.48E-04

Supplementary Table 5: Prediction of hair traits

	% Phenotypic variation explained			
**Trait**	**Age+Sex**	**Genetic PCs**	**Index SNPs + BLUP**	**Total**
Hair Shape	0.41	9.70	14.96	25.07
Beard	10.68[a]	12.54	17.53	40.75
Eyebrow	2.44[a]	3.69	16.30	22.43
Monobrow	0.22[a]	5.51	10.45	16.18
Hair Color	1.31	18.38	20.16	39.85
Hair Graying	19.51	1.91	6.62	28.04
Balding	13.16	5.37	8.77	27.30

[a] Sex was not included as a covariate for facial traits as these were assessed only in males.

While the total prediction values can have a similar interpretation to the heritability estimates, these are not directly numerically comparable for several reasons:

i) While h^2 is usually an upper bound for R^2, with typical sample sizes we expect prediction R^2 to be relatively lower (Makowsky et al. 2011), because of the difficulty in accurately estimating effect sizes for many SNPs in the BLUP computation as compared to estimating a few variance components for heritability.

ii) Heritability is calculated on the whole dataset while prediction scores are calculated in test subsets which are different from the training subsets used to build the models (Hastie et al. 2009). Since the test set on which prediction accuracy is calculated is different from the training set that produces the prediction model, the accuracy is likely to be smaller in magnitude in the test set than in the training set. E.g. the training R^2 for hair color was 100%, matching the observed heritability values.

iii) Prediction captures the proportion of phenotypic variation explainable only by the index SNPs + BLUP component, in contrast to the genome-wide kinship matrix used in heritability (Wray et al. 2013). The genome-wide kinship matrix used in heritability is thus more likely to capture polygenic effects from many genes with smaller effect sizes.

iv) The mathematical formulae are slightly different, e.g. while obtaining fraction of variance explained during calculation of heritability, the variance component due to covariates is removed first from the total phenotypic variance in the denominator (Yang et al. 2011).

Supplementary Table 6: SNPs showing suggestive association with hair traits

Table 1 presented SNPs showing genome-wide significant association with hair traits. SNPs with *P* values below the genome-wide significant threshold (5×10^{-8}) but above the suggestive significant threshold (10^{-5}) are presented here. If a genomic region has several associated SNPs, only the SNP with lowest P-value is shown. Annotations regarding SNP type (for intragenic SNPs), genes in the region (±300 KB from the SNP) and potential regulatory role were obtained from the UCSC Genome Browser. This list includes the two suggestively significant associations of the EDAR rs3827760 SNP with eyebrow thickness and Monobrow, as mentioned in the main text. A dot indicates that no relevant information is available for that SNP.

CHR	SNP	BP	P	Trait	SNP type	Genes in region	Regulatory annotation
4	rs4864363	137691750	4.38E-07	Hair Shape	.	.	.
7	rs73199888	109757018	1.52E-07	Hair Shape	.	EIF3IP1(+156.7kb)	.
10	rs4881147	3342774	2.99E-06	Hair Shape	.	PFKP(+163.8kb)| PITRM1(+127.7kb)| PITRM1-AS1(+152kb)	.
14	rs3742377	100166228	1.40E-07	Hair Shape	intron	CCDC85C(+95.5kb)| CCNK(+188.4kb)| CYP46A1| EML1(-93.52kb)| HHIPL1(+23.22kb)| MIR5698(+25.99kb)| SETD3(+219kb)	.
18	rs4800451	20716805	5.57E-06	Hair Shape	intron	CABLES1| MIR4741(+203.4kb)| RBBP8(+110.4kb)| TMEM241(-159.2kb)	Promoter
1	rs11121667	11038476	3.76E-07	Beard thickness	intron	ANGPTL7(-210.9kb)| C1orf127| CASZ1(+181.7kb)| EXOSC10(-88.2kb)| MASP2(-48.1kb)| MTOR(-128.1kb)| MTOR-AS1(-165.5kb)| SRM(-76.17kb)| TARDBP(-34.2kb)| UBIAD1(-294.8kb)	.
1	rs6684877	39557810	8.18E-06	Beard thickness	intron	AKIRIN1(+86.07kb)| GJA9(+210.5kb)| GJA9-MYCBP(+210.5kb)| MACF1| MYCBP(+218.8kb)| NDUFS5(+57.5kb)| RHBDL2(+150.4kb)| RRAGC(+232.3kb)	CTCF_Binding_Site| Enhancer
5	rs1003136	127347815	1.10E-06	Beard thickness	.	FBN2(-245.8kb)| LINC01184(-9.428kb)| SLC12A2(-71.67kb)	.
10	rs10788096	122242293	1.49E-06	Beard thickness	intron	C10orf85(-115.2kb)| MIR5694(-102.3kb)| PPAPDC1A| WDR11-AS1(-	.

						279kb)	
1	rs10908366	38121934	6.51E-06	Eyebrow thickness	.	C1orf109(-25.31kb)| C1orf122(-151.5kb)| CDCA8(-36.14kb)| DNALI1(+89.48kb)| EPHA10(-59.71kb)| GNL2(+60.35kb)| INPP5B(-204.4kb)| LINC01137(+181.9kb)| MANEAL(-137.8kb)| MEAF6(+141.5kb)| MIR5581(+155.3kb)| MIR6732(+176kb)| MTF1(-153.3kb)| RSPO1(+21.34kb)| SNIP1(+102kb)| YRDC(-146.7kb)| ZC3H12A(+172kb)	.
2	rs3827760	108997262	1.16E-07	Eyebrow thickness	missense	GCC2(-68.31kb)| LIMS1(-153.5kb)| SULT1C2(+70.89kb)| SULT1C2P1(+27.01kb)| SULT1C3(+115.5kb)| SULT1C4	Enhancer
4	rs1868245	87848739	2.30E-06	Eyebrow thickness	intron	AFF1(-7.414kb)| C4orf36(+35.16kb)| KLHL8(-232.5kb)| LOC100506746| PTPN13(+112.4kb)| SLC10A6(+78.32kb)	Promoter_Flanking_Region
5	rs9654415	72544390	2.56E-07	Eyebrow thickness	.	BTF3(-249.9kb)| FCHO2(+158kb)| FOXD1(-197.7kb)| TMEM171(+116.7kb)| TMEM174(+73.42kb)	.
5	rs10515457	132650871	2.77E-06	Eyebrow thickness	intron	FSTL4| HSPA4(+210.2kb)| MIR1289-2(-112.4kb)| ZCCHC10(+288.6kb)	.
7	rs201287033	150823227	7.22E-06	Eyebrow thickness	intron	ABCB8(+78.36kb)| ABCF2(-81.7kb)| AGAP3| AOC1(+264.8kb)| ASB10(-49.56kb)| ASIC3(+73.38kb)| ATG9B(+101.6kb)| CDK5(+68.18kb)| CHPF2(-106.3kb)| FASTK(+45.26kb)| GBX1(-22.45kb)| KCNH2(+147.8kb)| MIR671(-112.3kb)| NOS3(+111.5kb)| NUB1(-215.6kb)| SLC4A2(+49.61kb)| SMARCD3(-112.8kb)| TMUB1(+42.61kb)| WDR86(-255kb)| WDR86-AS1(-283kb)	Promoter
9	rs62578126	129375338	3.37E-06	Eyebrow thickness	.	LMX1B(-1.383kb)| MVB12B(+106kb)| NRON(+202.6kb)| ZBTB34(-247.6kb)| ZBTB43(-191.9kb)	TF_binding_site
15	rs112386516	78375229	1.48E-06	Eyebrow	.	ACSBG1(-87.96kb)| CIB2(-21.72kb)| CRABP1(-257.4kb)| DNAJA4(-181.3kb)|	Promoter_Fla

				thickness		IDH3A(-66.49kb)| LOC91450(+88.66kb)| LOC645752(+156kb)| MIR5003(-0.645kb)| SH2D7(-9.697kb)| TBC1D2B(+5.235kb)| WDR61(-200.3kb)	nking_Region
16	rs12597422	53887738	2.95E-06	Eyebrow thickness	intron	FTO| FTO-IT1(-185.6kb)| RPGRIP1L(+150kb)	.
2	rs3827760	109513601	1.46E-07	Monobrow	missense	CCDC138(+20.75kb)| EDAR| LIMS1(+209.9kb)| MIR4265(-244.3kb)| RANBP2(+111.3kb)| SH3RF3(-232.4kb)| SH3RF3-AS1(-230.2kb)	.
4	rs724818	121627113	4.16E-06	Monobrow	intron	PRDM5	.
5	rs7702331	72551134	4.28E-07	Monobrow	.	ANKRA2(-296.9kb)| BTF3(-243.1kb)| FCHO2(+164.8kb)| FOXD1(-191kb)| TMEM171(+123.5kb)| TMEM174(+80.16kb)	.
6	rs12197419	1709037	1.74E-06	Monobrow	intron	FOXC1(+94.91kb)| GMDS	.
9	rs12236890	3157137	2.02E-06	Monobrow	.	RFX3(-61.16kb)	.
14	rs1187437	54704459	3.51E-07	Monobrow	.	BMP4(+280.9kb)| CDKN3(-159.2kb)| CGRRF1(-272.1kb)| CNIH1(-189.2kb)| GMFB(-236.7kb)| MIR5580(+289.3kb)	.
2	rs213546	234739870	6.72E-06	Hair Graying	intron	DNAJB3(+87.21kb)| HJURP(-5.476kb)| LOC100286922(+75.88kb)| MROH2A| MSL3P1(-34.22kb)| SPP2(-219.5kb)| TRPM8(-86.17kb)| UGT1A1(+57.92kb)| UGT1A3(+57.92kb)| UGT1A4(+57.92kb)| UGT1A5(+57.92kb)| UGT1A6(+57.92kb)| UGT1A7(+57.92kb)| UGT1A8(+57.92kb)| UGT1A9(+57.92kb)| UGT1A10(+57.92kb)| USP40(+265.6kb)	.
4	rs2085601	89895944	9.01E-06	Hair Graying	intron	FAM13A| FAM13A-AS1(+244.7kb)| GPRIN3(-269.5kb)| HERC3(+266.3kb)| NAP1L5(+276.9kb)| TIGD2(-138kb)	.
8	rs7009516	24208847	4.26E-06	Hair Graying	intron	ADAM7(-89.66kb)| ADAM28| ADAMDEC1(-32.95kb)	Enhancer

11	rs1912702	79173082	1.82E-06	Hair Graying	.	MIR708(+59.93kb)| MIR5579(+39.81kb)| TENM4(+21.39kb)	.
14	rs11621135	71659609	5.92E-06	Hair Graying	.	LOC145474(-295kb)| PCNX(+77.51kb)| SNORD56B(-205.4kb)	.
15	rs281229	47718455	2.71E-07	Hair Graying	intron	SEMA6D	.
22	rs9626711	47686103	4.67E-06	Hair Graying	.	LOC101927722(-170.9kb)| TBC1D22A(+114.8kb)	.
3	rs9820421	188949286	3.24E-06	Hair Color	intron	TPRG1| TPRG1-AS2(-7.188kb)	.
9	rs58680346	7604965	1.78E-06	Hair Color	.	TMEM261(-191.5kb)	.
10	rs12355139	20565765	5.47E-06	Hair Color	intron	MIR4675(-275.1kb)| PLXDC2	Promoter_Flanking_Region
16	rs4782309	88721538	1.28E-07	Hair Color	.	APRT(-154.3kb)| CBFA2T3(-219.7kb)| CDT1(-148.6kb)| CTU2(-51.35kb)| CYBA(+4.046kb)| GALNS(-158.6kb)| IL17C(+14.66kb)| LOC339059(-87.64kb)| LOC100129697(-284.9kb)| LOC100289580(-76.05kb)| MIR4722(-61.15kb)| MIR5189(+186.1kb)| MVD| PABPN1L(-208.2kb)| PIEZO1(-60.21kb)| RNF166(-41.36kb)| SNAI3(-22.55kb)| SNAI3-AS1(-8.242kb)| TRAPPC2L(-202kb)| ZC3H18(+23.17kb)| ZFPM1(+120kb)| ZNF469(+214.4kb)	.
18	rs9949121	42306884	5.70E-06	Hair Color	intron	LOC101927921(+195.2kb)| MIR4319(-243.2kb)| SETBP1	.
23	rs5971087	23285900	4.93E-07	Hair Color	intron	DDX53(+265.7kb)| PTCHD1(-67.08kb)| PTCHD1-AS	.

Supplementary Table 7: Continental allele frequencies of index SNPs

Allele frequencies are provided for all index SNPs listed in Table 1. CEU, YRI, CHB are Europeans, Yoruba and Chinese from the 1000 genomes project. NAM are Native Americans and CAN is the CANDELA sample examined here. NAM data are from populations included in Reich et al. (2012).

Trait	Region	SNP	Closest gene	Alleles	Derived Allele Frequency (%)

				Ancestral>derived	CEU	YRI	CHB	NAM	CAN
Hair Shape	1q21	rs11803731	TCHH	A>T	23	0	0	0	9
Hair Shape	2q12	rs3827760	EDAR	A>G	0	0	94	98	42
Hair Shape	10p14	rs17143387	GATA3	T>G	16	4	52	73	41
Hair Shape	16p11	rs11150606	PRSS53	T>C	4	0	83	47	23
Beard thickness	2q12	rs365060	EDAR	G>C	93	35	2	2	51
Beard thickness	4q12	rs4864809	LNX1	A>G	61	42	41	60	61
Beard thickness	6q21	rs6901317	PREP	G>T	35	8	66	57	42
Beard thickness	7q31	rs117717824	FOXP2	G>T	0	0	5	13	5
Eyebrow thickness	3q22	rs112458845	FOXL2	G>A	99	68	96	36	72
Monobrow	2q36	rs2218065	PAX3	A>G	64	34	38	25	42
Hair Graying	6p25	rs12203592	IRF4	C>T	16	0	0	0	6
Hair Color	5p13	rs183671	SLC45A2	T>G	98	17	8	5	50
Hair Color	6p25	rs12203592	IRF4	C>T	16	0	0	0	6
Hair Color	11q14	rs598952	TYR	T>A	28	74	74	95	57
Hair Color	15q13	rs12913832	HERC2/OCA2	A>G	77	0	0	1	23
Hair Color	15q21	rs1426654	SLC24A5	G>A	100	1	3	1	56
Balding	10q22	rs2814331	GRID1	C>T	85	93	41	96	90
Balding	Xq12	rs4258142	AR/EDA2R	C>T	85	0	100	99	88

SUPPLEMENTARY REFERENCE

Wray, N.R. et al. (2013). Pitfalls of predicting complex traits from SNPs. Nat Rev Genet 14, 507-15.

Makowsky, R. et al. (2011). Beyond missing heritability: prediction of complex traits. PLoS Genet 7, e1002051.

Yang, J., Lee, S.H., Goddard, M.E. & Visscher, P.M. (2011). GCTA: a tool for genome-wide complex trait analysis. Am J Hum Genet 88, 76-82.

Hastie, T., Tibshirani, R. & Friedman, J.H. (2009). The elements of statistical learning: data mining, inference, and prediction, xxii, 745 p. (Springer, New York, NY, 2009).

Reich, D. et al. (2012), Reconstructing Native American population history. Nature 488, 370–374.
